# Frequency and Spectrum of Actionable Secondary Findings in the Maltese Population

**DOI:** 10.1002/mgg3.70143

**Published:** 2025-09-19

**Authors:** Laura Grech, Celine Ann Grech, Jasmine Spiteri, Dillon Mintoff, Nikolai Paul Pace

**Affiliations:** ^1^ Department of Applied Biomedical Sciences, Faculty of Health Sciences University of Malta Msida Malta; ^2^ Centre for Molecular Medicine and Biobanking University of Malta Msida Malta; ^3^ Department of Anatomy, Faculty of Medicine and Surgery University of Malta Msida Malta; ^4^ Department of Pathology, Faculty of Medicine and Surgery University of Malta Msida Malta

**Keywords:** ACMG SF v3.2, exome sequencing, genomic medicine, secondary findings

## Abstract

**Background:**

The identification of actionable secondary findings (SFs) through clinical exome sequencing has become increasingly relevant with the integration of genomics into routine healthcare. The frequency and spectrum of these findings vary across populations.

**Methods:**

We analyzed exome sequencing data from 350 unrelated Maltese individuals, comprising 320 pseudonymised controls and 30 participants from the pilot sequencing phase of the national biobank DwarnaBio, to assess the prevalence of pathogenic or likely pathogenic (P/LP) variants in the ACMG SF v3.2 gene list. All samples underwent uniform sequencing, rigorous quality control, and variant interpretation according to ACMG/AMP guidelines.

**Results:**

Actionable P/LP variants were identified in 12 individuals (3.4%) across autosomal dominant genes, predominantly associated with inherited cardiac conditions and cancer predisposition syndromes. These findings highlight the importance of including underrepresented populations in genomic research and emphasize the need to establish provisions for the return of clinically actionable results to biobank participants, supported by access to genetic counseling.

**Conclusion:**

Our results advocate for the integration of population‐specific genomic data into national precision medicine frameworks, particularly for small or isolated populations where tailored approaches to variant curation and clinical translation are required. This study provides the first baseline estimate of actionable SFs in the Maltese population and offers insights for advancing precision medicine frameworks.

## Graphical abstract

In a populaiton cohort of 350 individuals, actionable P/LP variants were identified in 12 individuals (3.4%) across autosomal dominant genes.

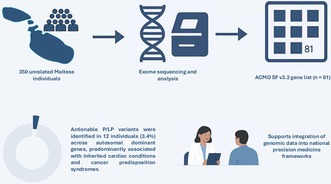

## 1 Introduction

The rapid advancement and widespread adoption of next‐generation sequencing technologies, particularly whole exome and whole genome sequencing, have revolutionized clinical diagnosis. As these methods become increasingly integrated within mainstream healthcare, the identification of secondary findings (SFs)—defined as pathogenic or likely pathogenic (P/LP) variants in genes that are unrelated to the primary indication for testing—has emerged as a significant clinical and ethical concern. SFs involve genes with established medical actionability where evidence‐based interventions for disease prevention, surveillance, or treatment are available. Consequently, the identification of SFs holds potential implications not only for individual patient care but also for cascade testing and family planning (Green et al. 2013).

To standardize the reporting of SFs, the American College of Medical Genetics and Genomics (ACMG) curates a list of defined genes recommended for disclosure (Kalia et al. 2017; Scheuner et al. 2015). The list is periodically updated, with the latest version (ACMG SF v3.2) including 81 loci associated with actionable conditions (Miller et al. 2023). However, the frequency of actionable SFs demonstrates considerable variability across populations and studies, owing to differences in ascertainment strategies, gene selection, and variant curation methodologies (Amendola et al. 2015; Backman et al. 2021; Dewey et al. 2016; Kuo et al. 2020; Yang et al. 2014). The disease spectrum of SFs also contrasts across populations, with differing predominance of cancer‐susceptibility genes and cardiovascular disease genes reported between studies (Gordon et al. 2020; Jalkh et al. 2020).

The analysis of population‐scale genome or exome sequences is a powerful approach that provides insight into the genetic epidemiology of SFs, quantifying their prevalence and informing the design and implementation of public health programs and healthcare policies (Elfatih et al. 2024). To date, most large‐scale SF burden studies have disproportionately prioritized populations of Northern European ancestry, with disparate underrepresentation of global demographic makeup. The reported heterogeneity in SF frequency further underscores the need to establish population‐specific frequencies, particularly in understudied populations, to better reflect diversity. The lack of population diversity in genomic research has public health implications, as it can impact on the translation of genomic findings, increase the likelihood of returning variants of uncertain significance, and lead to disparities in genetic counselling experiences and outcomes (Fatumo et al. 2022; Montgomery et al. 2025), thereby limiting the generalizability of genomic medicine.

The Maltese archipelago located in the Southern Mediterranean is home to approximately 400,000 ethnically Maltese inhabitants. This population is characterized by unique genetic attributes, including a propensity for founder effects (Mintoff et al. 2023; Spiteri et al. 2025) and evidence of admixture with European and Middle Eastern datasets (Gilbert et al. 2022). Furthermore, the Maltese exhibit a high prevalence of obesity and cardiometabolic disease, along with differing risk allele profiles for complex traits (Schembri, Pace, Degenhardt, et al. 2019; Schembri, Pace, Vella, et al. 2019). Despite these factors, there is a paucity of population‐specific genomic data, and gaps remain in both genomic literacy and the implementation of precision medicine (Borg et al. 2024; Mintoff et al. 2024).

Against this background, we sought to characterize the frequency and spectrum of actionable P/LP SFs in the ACMG SF v3.2 list in a reference cohort of Maltese individuals unselected for clinical disease. As a secondary objective, we also explore the carrier frequency for P/LP variants in the ACMG gene list, thereby providing preliminary epidemiological data for genomic medicine in this population.

## 2 Materials and Methods

### 2.1 Ethics Statement

This study was approved by the institutional ethics review board of the University of Malta (CMMB‐2023‐00004), and the study protocol complied with the Declaration of Helsinki. All participants provided written informed consent for both study participation and genetic analysis.

### 2.2 Study Population

To assess the frequency and spectrum of actionable SFs in the Maltese population, we analyzed two whole exome sequencing (WES) datasets. The first dataset comprised sequences derived from 320 unrelated adults with self‐reported third‐generation Maltese–Caucasian ancestry. This pseudonymized dataset was assembled through a combination of convenience and random sampling and served as a reference control cohort for selected genomic studies in the Maltese population (Mintoff et al. 2023; Spiteri et al. 2025). All samples were pseudonymized prior to sequencing and analysis, with no retention of identifiable information. Since participants were not consented for recontact, return of genetic results, or cross‐linking to health records, this dataset is used solely as an ethnically matched cohort for estimating population‐level allele frequencies and interpreting genetic findings from case cohorts.

The second cohort comprised 30 WES sequences derived from the pilot analysis of DwarnaBio, the recently established national population biobank in Malta. DwarnaBio aims to provide a centralized, population‐based repository of biospecimens and associated clinical data, with consent for recontact, multi‐omic research, and longitudinal follow‐up. DwarnaBio operates a dynamic consent system via an online platform (dwarna.mt), which prioritizes participant engagement to foster trust and public involvement in research (Mamo et al. 2020). DwarnaBio follows international biobanking standards, facilitating interoperability and data sharing within the BBMRI‐ERIC network, of which it is a member.

In the aggregate analysis (*n* = 350), participants ranged from 18 to 57 years of age at the time of recruitment, with a balanced sex distribution (48% female). Recruitment aimed to capture a cross section of the ostensibly healthy general adult population and was unselected for any specific disease phenotype. All individuals underwent clinical characterization at recruitment. At this stage, participants with a known genetic diagnosis or syndrome, a history of consanguinity, or close familial relationships were excluded. Individuals with intellectual disabilities, developmental delay, or those unable to provide informed consent were also excluded. Importantly, no participant with a previously known genetic diagnosis or syndromic condition was included in this analysis. The exclusion of individuals with preexisting genetic diagnoses specifically prevents ascertainment bias, ensuring the cohort reflects the baseline prevalence of actionable SFs in an unselected population. This approach aligns with the primary objective of establishing population‐level genomic estimates for a national biobank.

### 2.3 Exome Sequencing, Data Processing and Bioinformatic Analysis

Genomic DNA was extracted from peripheral blood leukocytes collected in K_2_‐EDTA anticoagulated blood using a QIAmp DNA Blood Mini Kit (Qiagen Inc., CA, USA). DNA concentration and integrity were assessed using Qubit 3.0 fluorimetry (ThermoFisher Scientific) and agarose gel electrophoresis.

WES libraries were prepared using the SureSelect Human All Exon V8 capture design (Agilent Technologies), targeting the protein‐coding regions defined by RefSeq, CCDS and GENCODE. Each library was prepared from 300 ng of DNA according to the manufacturer's protocols involving sonication, end‐repair, ligation‐mediated PCR (LM‐PCR) amplification, adapter ligation, hybridisation and capture by streptavidin‐modified nano‐magnetic beads, followed by library quality control using qPCR. The resulting libraries were sequenced in 150 bp paired end reads on a NovaSeq6000 sequencer (Illumina Inc.).

Sequencing data were analyzed using the following bioinformatic pipeline. Image analyses were performed with the default parameters of Illumina RTA pipeline. FASTQ files underwent quality assessment with FastQC and were mapped and aligned to the Human Reference Genome (GRCh37) using the Burrows–Wheeler transformation algorithm (Li and Durbin 2009). Duplicated reads were removed using Picard (https://broadinstitute.github.io/picard/). GATK was used to perform indel realignment, base recalibration, and SNV/InDel calling according to GATK best practice guidelines. Variant annotation was performed using ANNOVAR. The following sequencing quality metrics were obtained: Q30 scores of 94.77%, average mapping efficiency to the reference genome was about 98.79%, and the average depth of the target regions was about 78.90×. Depth metrics were calculated using *mosdepth* v0.3.8 using the Agilent V8 target BED file, following duplicate removal (Pedersen and Quinlan 2018). Across the 350 exome libraries, a median 95.3% (range 94.2%–97.4%) of target bases were sequenced to a depth of ≥ 20×. The sequenced libraries met the ≥ 90% threshold recommended for clinical‐grade heterozygous variant detection.

Variant analysis was restricted to all the 81 genes included in the revised ACMG guidelines for reporting of SFs in clinical exome and genome sequencing (ACMG SFv3.2) (Miller et al. 2023) as detailed in Table S1. Variants were retained if they affected protein coding regions (missense, nonsense, frameshift, non‐frameshift indels, or splice site/region variants) and excluded if located in noncoding UTRs or deep intronic regions (>±10 bp from exon boundaries). Variant quality control was performed to retain high‐confidence variant calls for downstream analysis, and the following VQSR thresholds were applied: genotype quality (GQ) ≥ 20, read depth (DP) ≥ 10, allele balance (AB) ≥ 0.2 for heterozygous genotypes, mapping quality (MQ) ≥ 40, strand bias filter: FisherStrand (FS) < 60 for SNPs, FS < 200 for indels. Annotation was limited to variants mapping to canonical transcripts as defined by MANE Select (v1.4). Pathogenicity was classified according to ACMG/AMP guidelines using the Franklin (v.80) variant interpretation platform (Genoox, Tel Aviv, Israel) (Richards et al. 2015). Interpretation of predicted loss‐of‐function (PVS1) variants and computational evidence (PP3/BP4) for missense variants followed expert recommendations (Pejaver et al. 2022; Tayoun et al. 2018). Allele frequencies for shortlisted variants were derived from GnomAD v4.1.0. Only variants classified as P/LP were retained, while variants of uncertain significance were excluded. For shortlisted variants, additional annotation included cross‐referencing ClinVar entries (ClinVar Release 2025‐01) and integrating scores from in silico predictors of pathogenicity and conservation (CADD, EVE, ESM‐1b, and AlphaMissense) (Frazer et al. 2021; Rives et al. 2021). Repositories were accessed in January–February 2025.

## 3 Results

In this study, we analyzed a population‐scale dataset of Maltese individuals to assess the prevalence and spectrum of P/LP variants in the 81 medically actionable genes in the ACMG SF v3.2 list.

Of the 350 individuals, 12 (3.4%) harbored a P/LP medically actionable variant in one of nine autosomal dominant (AD) genes (Table 1). No individuals carried biallelic variants in autosomal recessive genes, or were hemizygous, heterozygous, or homozygous for X‐linked P/LP variants. With regard to disease category, seven individuals had a P/LP variant in five genes associated with inherited cardiac disease, three individuals had variants in cancer‐related genes, and two individuals had P/LP variants in *RYR1* (miscellaneous phenotypes). No P/LP variants were detected in genes related to inborn errors of metabolism.

**TABLE 1:** Details of the autosomal dominant variants in the ACMG SF v3.2 gene list showing the residue change, frequency in GnomAD database (non‐Finnish Europeans), ACMG classification and criteria and ClinVar accession entry.

Gene	Transcript	Nucleotide	AA change	Exon	dbSNP	ACMG‐AMP classification	ClinVar entry	GnomAD NFE
*APC*	NM_000038.6	c.3920T>A	p.Ile1307Lys	16	rs1801155	LP—PP5, PM2	VCV000000822.99	0.0006364
*BRCA2*	NM_000059.4	c.5073dupA	p.Trp1692fs	11	rs1265028174	P—PVS1, PM2, PS4, PP5	VCV000037943.107	0.000028
*DES*	NM_001927.4	c.1048C>T	p.Arg350Trp	6	rs62636492	P—PS4, PM2, PM5, PM1, PP3, PP2, PP5	VCV000044244.19	0.00002639
*FBN1*	NM_000138.5	c.494G>A	p.Arg165Gln	6	rs1060501079	LP—PM2, PM1, PS4, PP2, PM5	VCV000807252.17	0.00001759
*KCNQ1*	NM_000218.3	c.1189C>T	p.Arg397Trp	9	rs199472776	LP—PM2, PP5, PP3, PP2	VCV000052970.56	0.0003021
*MSH2*	NM_000251.3	c.1021C>G	p.Leu341Val	6	rs748115066	LP—PP3, PM2, PM5, BP6	VCV000232192.24	0.000008792
*MYBPC3*	NM_000256.3	c.1591G>C	p.Gly531Arg	17	rs397515912	P—PS1, PP3, PM2, PS4, PM5, PS3, PP1, PP5	VCV000042550.56	0.00003149
*PTEN*	NM_000314.8	c.148_149delAT	p.Ile50Ter	2		LP—PVS1, PM2		
*RYR1*	NM_000540.3	c.1589G>A	p.Arg530His	15	rs111888148	P—PM3, PP1, PS3,PM1, PP2,PM2, PM5, PP3	VCV000133101.54	0.00005274
*RYR1*	NM_000540.3	c.10501G>T	p.Asp3501Tyr	71	rs763259167	LP—PP3, PM2, PS4, PP2, PP5	VCV001498791.17	0.000008799
*TGFBR2*	NM_003242.6	c.383dupA	p.Pro129fs	3	rs79375991	LP—PVS1, PM2	VCV000662321.16	0.0006371

In the cardiovascular disease category, two individuals were heterozygous for the *DES* p.Arg350Trp pathogenic variant implicated in dilated cardiomyopathy (DCM). One individual was heterozygous for the *MYBPC3* p.Gly531Arg pathogenic variant associated with hypertrophic cardiomyopathy (HCM), and two individuals were heterozygous for the *KCNQ1* p.Arg397Trp (LP) variant implicated in long QT syndrome (LQTS). One individual was heterozygous for the *TGFBR2* p.Pro129fs (LP) variant associated with Loeys–Dietz syndrome, while a LP variant related to Marfan Syndrome (*FBN1* p.Arg165Gln) was detected in another individual. No P/LP variants were identified in the two X‐linked genes on the ACMG list.

With regard to cancer‐associated loci, three unique P/LP variants were observed in the heterozygous state. These included: *BRCA2* p.Trp1692fs (P) associated with hereditary breast and ovarian cancer, *MSH2* p.Leu341Val (LP) associated with Lynch syndrome, and the novel *PTEN* p.Ile50Ter (LP) associated with Cowden syndrome and glioma susceptibility. Two other individuals were heterozygous for a medically actionable variant in *RYR1* (NM_000540.3). One individual was heterozygous for the pathogenic variant p.Arg530His, and the other for the LP p.Asp3501Tyr variant. In silico pathogenicity predictors for missense variants are summarized in Table S2.

In addition to the dominant disease alleles, we also identified 21 individuals (6%) who were carriers of a high‐risk disease allele in four AR actionable genes (Table 2). P/LP variants were identified in *MUTYH* (14 individuals) and *ATP7B* (5 individuals), known to cause MUTYH‐Associated Polyposis and Wilson disease respectively when present in the compound heterozygous or homozygous state. A monoallelic P/LP variant in *GAA*, associated with Pompe disease, and the *HFE* p.Cys282Tyr variant, associated with hereditary hemochromatosis, were also detected.

**TABLE 2:** Details of the autosomal recessive variants in the ACMG SF v3.2 gene list showing the residue change, frequency in GnomAD database (non‐Finnish Europeans), ACMG classification and criteria, and ClinVar accession entry.

Gene	Transcript	Nucleotide	AA change	Exon	dbSNP	ACMG‐AMP classification	ClinVar	GnomAD NFE
*ATP7B*	NM_000053.4	c.2605G>A	p.Gly869Arg	11	rs191312027	P—PM3, PM2, PM1, PP3, PM5, PP2, PP5	VCV000157939.86	0.00125
*ATP7B*	NM_000053.4	c.3207C>A	p.His1069Gln	14	rs76151636	P—PM3, PM2, PM5, PM1, PP3, PP3, PS3 PP1, PP5	VCV000003848.97	0.0015
*ATP7B*	NM_000053.4	c.3278A>G	p.Asp1093Gly	15	rs1022670769	LP—PM2, PM1, PP3, PP2	VCV000857095.11	0
*ATP7B*	NM_000053.4	c.3784G>T	p.Val1262Phe	18	rs769484789	P—PP2, PS3, PP5	VCV000550902.15	0.000008827
*GAA*	NM_000152.5	c.1210G>C	p.Asp404His	8		LP—PP3, PM2, PM5, PM1, PP2		
*HFE*	NM_000410.4	c.845G>A	p.Cys282Tyr	4	rs1800562	LP—PS3, PP3, PP5	VCV000000009.124	0.05766
*MUTYH*	NM_001048174.2	c.737G>A	p.Arg246Gln	10	rs149866955	LP—PM2, PM5, PP3, PM1, PP5	VCV000041764.51	0.000351
*MUTYH*	NM_001048174.2	c.1103G>A	p.Gly368Asp	13	rs36053993	LP—PP3, PM2, PM5, PP5	VCV000005294.125	0.004894
*MUTYH*	NM_001048174.2	c.1143_1144dupGG	p.Glu382fs	13	rs587780078	P—PVS1, PM3, PM2, PS3, PP1, PP5	VCV000127831.52	0.00004656
*MUTYH*	NM_001048174.2	c.1353_1355del	p.Glu452del	14	rs587778541	LP—PM2, PM4, PS4, PP5	VCV000127838.84	0.0001319

In addition, we identified five individuals who carried the *APC* p.Ile1307Lys variant, a low‐penetrance risk allele predominantly observed in individuals of Ashkenazi Jewish ancestry. This variant is associated with a modestly increased risk of colorectal cancer in this population. Carriers of this variant do not exhibit clinical features of Familial adenomatous polyposis (FAP), distinguishing it from higher penetrance *APC* variants (Forkosh et al. 2022). The *APC* p.Ile1307Lys variant is considered actionable in the Ashkenazi population due to its established association with colorectal cancer risk, yet current evidence does not support its inclusion in actionable screening panels for other populations (Valle et al. 2023).

**FIGURE 1:**
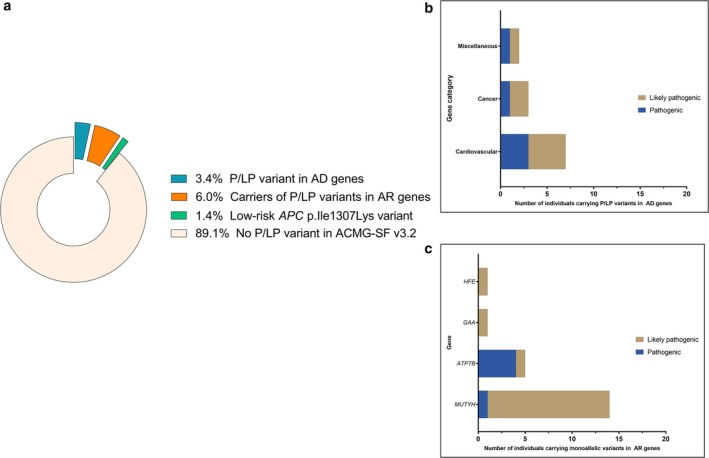
(a) Distribution and classification of pathogenic/likely pathogenic (P/LP) variants identified in ACMG SF v3.2 genes. Donut chart showing the overall proportion of individuals in each variant category: 3.4% carried a P/LP variant in autosomal dominant (AD) genes, 6.0% were monoallelic carriers of P/LP variants in autosomal recessive (AR) genes, and 1.4% carried the low‐risk *APC*:p.Ile1307Lys variant. (b) Distribution of P/LP variants in AD genes by gene category and clinical classification. (c) Distribution of monoallelic P/LP variants in AR genes by gene and clinical classification. Bars represent the number of individuals with variants categorized as either likely pathogenic or pathogenic.

A graphical summary of the salient findings is provided in Figure 1.

## 4 Discussion

In this report, we determine the frequency and spectrum of actionable pathogenic SFs in a cohort of 350 unrelated Maltese individuals using exome sequencing data. By applying the ACMG SF v3.2 list of 81 genes, we identified P/LP variants in 3.4% of participants, a frequency congruent with estimates from Qatari (3.5%) and East Asian cohorts (3.4%) but higher than those reported in European (2.8%), Lebanese (2.1%), and Taiwanese (1.8%) populations (Chan et al. 2022; Jalkh et al. 2020; Kuo et al. 2020; Van Hout et al. 2020). We also report the carrier frequency of deleterious variants in recessive disease genes within this panel. Our findings are relevant as they provide the first population‐level insight into the burden of actionable SFs in the Maltese population—a Southern European island underrepresented in large‐scale genomic studies.

Our results warrant interpretation in the context of population genetics. The genetic heritage of contemporary Maltese derives from the various populations that have colonized, traded, or settled in the archipelago. The collective genetic profile in the Maltese supports a genetic isolate with differences in genetic architecture from the mainland European average, with genetic founders being frequently described in this population (Borg et al. 2021; Gilbert et al. 2022; Mintoff et al. 2023; Spiteri et al. 2025). It is acknowledged that several large‐scale studies have estimated the prevalence of SFs derived from population‐based genome sequencing initiatives, predominantly focusing on cohorts of European and North American ancestry. However, certain demographic groups remain underrepresented in global genomic research endeavors. In particular, the underrepresentation of Arab genomes with historical ties to Malta, such as Algeria and Libya, is relevant (Bhattacharya et al. 2023), and while the addition of Qatari Biobank data is a great advance, it is not representative of the North African and Levantine countries (Mbarek and Ismail 2022). The growing imperative to develop and expand population‐specific datasets to advance precision medicine underscores the necessity of addressing this critical gap (Backman et al. 2021). The lack of diversity in genomic research also impedes the optimal development of genomic medicine programs, perpetuates inequities, and has been associated with diagnostic challenges (Serrano et al. 2023).

While several studies have reported the frequency of SFs across diverse populations, they reveal significant variability, with estimates ranging from 0.59% to as high as 17% (Jain et al. 2018; Rego et al. 2018). This variability can be attributed to differences in cohort characteristics, with study designs ranging from those based on healthy adults to specific patient groups (Elfatih et al. 2024; Zakeri et al. 2021). Furthermore, differences in sequencing methodologies, variant filtering pipelines, and the composition of the gene panel analyzed are also relevant. These methodological inconsistencies have been systematically reviewed and remain a challenge in efforts to harmonize global SF data (Elfatih et al. 2021).

Beyond the overall frequency, the molecular spectrum of variants identified is clinically significant. Our study identified individuals with actionable deleterious variants implicated in hereditary breast and ovarian cancers, colorectal cancers, cardiomyopathies, arrhythmias, and aortopathies. The clinical implications of these findings are significant. Identifying individuals with medically actionable variants enables early monitoring and possible preventive treatment, potentially mitigating the risk of severe health outcomes (Haer‐Wigman et al. 2019). For instance, individuals with cardiac disease‐related variants may benefit from regular cardiovascular monitoring and preventive measures, while those with cancer predisposition variants could undergo more frequent screenings and adopt lifestyle modifications to reduce their risk (Ghodeshwar et al. 2023). The identification of recurrent variants within the cohort may suggest the presence of population‐specific founder alleles, warranting further investigation through haplotype analysis and segregation studies.

The population‐level public health impact of SFs is undisputed, and thus their integration into health policy necessitates evidence‐based frameworks that balance clinical utility, ethical safeguards, and equitable implementation (Majeed et al. 2024; Wolf and Green 2023). Policy frameworks should prioritize tiered or dynamic consent models to empower research participants and minimize decisional burden. Mandatory clinical‐grade validation of research‐identified variants through accredited laboratories is essential to mitigate false positives before disclosure. Policies should guarantee universal access to genetic counseling to reduce disparities in interpretation and enable the development of region‐specific SF lists and screening, particularly for underrepresented groups. Failure to address these pillars through resource allocation and provider education risks undermining trust in genomic medicine and exacerbating healthcare disparities. Nevertheless, the identification of SFs presents psychological challenges for individuals and their families. Disclosure of medically actionable variants can cause emotional distress, create a sense of vulnerability, and impose decisional conflicts in patients and their families. For this reason, the ACMG now supports offering patients the choice to opt out of receiving SFs, acknowledging the complex personal, familial, and societal implications of receiving genomic risk information. Genetic counseling is therefore essential, not only to interpret the clinical implications of SFs but also to provide affected individuals the necessary support and guidance to cope with the potential emotional and psychological impact (Nambot et al. 2021).

Some limitations merit consideration. In addition to the limited number of subjects, the study design precluded phenotypic correlation, segregation studies within kindreds, and thus the capacity to explore and refine genotype–phenotype associations. Actionable SFs will eventually be returned to DwarnaBio biobank participants who explicitly opted into receiving such results during the consent process. Prior to disclosure, variants will undergo independent validation in a clinically accredited laboratory, followed by genetic counseling in accordance with DwarnaBio's governance framework and standard operating procedures for the return of genomic results.

Additionally, exome sequencing inherently omits deep intronic variants that modulate splicing activity and promoter variants, does not capture complex structural variants such as inversions or large deletions/duplications. Furthermore, the use of convenience sampling and the lack of stratified randomization methodology employed may introduce sampling bias, limiting the generalizability of these findings. Like other population biobanks, our dataset may reflect a “healthy volunteer” bias, with differing sociodemographic, physical, lifestyle, and health characteristics compared with nationally representative data sources (Fry et al. 2017). The integration of data from two sources, while collectively representative, may still reflect latent differences in ascertainment approaches and recruitment strategies. Although all samples were processed using an identical sequencing methodology and variant interpretation pipeline, the potential for batch effects or subtle differences in cohort characteristics cannot be excluded. These factors should be considered when interpreting aggregated variant frequencies and may warrant sensitivity analyses in future expanded datasets.

Additional caveats are shared with most SF prevalence studies. The reported frequency estimates in this study reflect the proportion of individuals harboring P/LP variants, but not the proportion of disease cases attributable to those variants. This requires access to genotyped clinical collections and is of relevance to characterizing potential founder variants that might be enriched in specific populations. Importantly, this study cannot infer variant penetrance as presumed pathogenic variants are often found in asymptomatic individuals (Gudmundsson et al. 2024). The true prevalence and penetrance of monogenic disease variants are often not known because of ascertainment bias in clinical cohorts. For example, studies have shown that pathogenic variants in the monogenic diabetes gene *HNF1A* demonstrate significantly lower penetrance in clinically unselected individuals compared to clinically referred probands (Mirshahi et al. 2022). This suggests that the clinical interpretation of deleterious variants may require contextualization based on the specific setting in which a pathogenic monogenic variant was identified.

Such considerations are increasingly pertinent in light of paradigm shifts in genomic medicine, including the growing accessibility of exome sequencing as a first‐tier diagnostic test and the emerging implications of newborn genomic sequencing initiatives (Ziegler et al. 2025). Furthermore, monogenic risk is modulated by an individual's polygenic background in conditions such as familial hypercholesterolemia, hereditary breast and ovarian cancer, and Lynch syndrome (Fahed et al. 2020). Indeed, polygenic risk alone can, in some cases, confer disease risk equivalent to that of penetrant monogenic mutations (Maamari et al. 2022).

## 5 Conclusion

This study provides the first population‐based estimate of medically actionable SFs in the Maltese population. Our results highlight the importance of including underrepresented populations in genomic research and contribute data to the equitable implementation of precision medicine initiatives. Future studies should focus on integrating findings with clinical data, expanding cohort sizes, and developing region‐specific strategies for variant interpretation and return of results. Our findings underscore the need to establish mechanisms for the ethical return of clinically actionable results to biobank participants, accompanied by access to genetic counseling services to support informed decision‐making. Moreover, integrating such genomic insights into national precision medicine frameworks is particularly relevant for small or isolated populations, where tailored variant interpretation pipelines and region‐specific screening guidelines can enhance the clinical utility and equity of genomic healthcare delivery.

## Author Contributions

Conceptualisation and design of study: N.P.P. and D.M.; Data curation and analysis: N.P.P. and L.G.; Writing, reviewing, and editing: D.M., C.A.G, J.S., and L.G.; Supervision: N.P.P. and D.M.

## Conflicts of Interest

The authors declare no conflicts of interest.

## Supporting information


**Table S1:** ACMG SF v3.2 list with respective disease associations.
**Table S2:** The in silico predicted impact of coding substitutions on protein stability and assessment of evolutionary conservation.

## Data Availability

The datasets used and/or analyzed during the current study are available from the corresponding author upon reasonable request.
