# Supplementary material for: Frequency and Spectrum of Actionable Secondary Findings in the Maltese Population
Source: Mol Genet Genomic Med. 2025 Sep 19;13(9):e70143. doi: 10.1002/mgg3.70143 (PMC12447238; doi:10.1002/mgg3.70143)
Supplement: Supplementary file 1 — Table S1: ACMG SF v3.2 list with respective disease associations. Table S2: The in silico predicted impact of coding substitutions on protein stability and assessment of evolutionary conservation. [file MGG3-13-e70143-s001.docx]

**Supplementary table 1: ACMG SF** **v3.2 list with respective disease associations**

| **Gene name** | **Gene stable ID** | **MIM morbid description** |
| --- | --- | --- |
| *ACTA2* | ENSG00000107796 | AORTIC ANEURYSM, FAMILIAL THORACIC 6; AAT6;;FAMILIAL THORACIC AORTIC ANEURYSM WITH LIVEDO RETICULARIS AND IRIS FLOCCULI |
| *ACTA2* | ENSG00000107796 | SMOOTH MUSCLE DYSFUNCTION SYNDROME; SMDYS;;MULTISYSTEMIC SMOOTH MUSCLE DYSFUNCTION SYNDROME; MSMDS;;MYDRIASIS, CONGENITAL, WITH PATENT DUCTUS ARTERIOSUS, THORACIC AORTIC ANEURYSM, AND VASCULOPATHY |
| *ACTA2* | ENSG00000107796 | MOYAMOYA DISEASE 5; MYMY5 |
| *ACTC1* | ENSG00000159251 | CARDIOMYOPATHY, FAMILIAL HYPERTROPHIC, 11; CMH11 |
| *ACTC1* | ENSG00000159251 | ATRIAL SEPTAL DEFECT 5; ASD5 |
| *ACTC1* | ENSG00000159251 | CARDIOMYOPATHY, DILATED, 1R; CMD1R LEFT VENTRICULAR NONCOMPACTION 4, INCLUDED; LVNC4, INCLUDED |
| *ACVRL1* | ENSG00000139567 | TELANGIECTASIA, HEREDITARY HEMORRHAGIC, TYPE 2; HHT2 PULMONARY ARTERIAL HYPERTENSION, HEREDITARY HEMORRHAGIC TELANGIECTASIA-RELATED, INCLUDED |
| *APC* | ENSG00000134982 | COLORECTAL CANCER; CRC;;COLON CANCER |
| *APC* | ENSG00000134982 | HEPATOCELLULAR CARCINOMA;;HCC;;CANCER, HEPATOCELLULAR;;LIVER CANCER;;LIVER CELL CARCINOMA; LCC;;HEPATOMA HEPATOBLASTOMA, INCLUDED;;HEPATOBLASTOMA CAUSED BY SOMATIC MUTATION, INCLUDED |
| *APC* | ENSG00000134982 | DESMOID DISEASE, HEREDITARY; DESMD;;FIBROMATOSIS, FAMILIAL INFILTRATIVE; FIF DESMOID TUMOR CAUSED BY SOMATIC MUTATION, INCLUDED |
| *APC* | ENSG00000134982 | FAMILIAL ADENOMATOUS POLYPOSIS 1; FAP1;;ADENOMATOUS POLYPOSIS OF THE COLON; APC;;FAMILIAL POLYPOSIS OF THE COLON; FPC;;POLYPOSIS, ADENOMATOUS INTESTINAL GARDNER SYNDROME, INCLUDED; GS, INCLUDED;;BRAIN TUMOR-POLYPOSIS SYNDROME 2, INCLUDED; BTPS2, INC /.../;FAMILIAL ADENOMATOUS POLYPOSIS, ATTENUATED, INCLUDED; AFAP, INCLUDED;;ADENOMATOUS POLYPOSIS COLI, ATTENUATED, INCLUDED; AAPC, INCLUDED;;ADENOMA, PERIAMPULLARY, SOMATIC, INCLUDED |
| *APC* | ENSG00000134982 | GASTRIC CANCER GASTRIC CANCER, INTESTINAL, INCLUDED |
| *APC* | ENSG00000134982 | GASTRIC ADENOCARCINOMA AND PROXIMAL POLYPOSIS OF THE STOMACH; GAPPS;;POLYPOSIS, GASTRIC; POLYPOSIS OF GASTRIC FUNDUS WITHOUT POLYPOSIS COLI;;FUNDIC GLAND POLYPOSIS |
| *APOB* | ENSG00000291544 | HYPERCHOLESTEROLEMIA, FAMILIAL, 2; FHCL2;;HYPERCHOLESTEROLEMIA, AUTOSOMAL DOMINANT, TYPE B;;APOLIPOPROTEIN B-100, FAMILIAL LIGAND-DEFECTIVE;;HYPERCHOLESTEROLEMIA, FAMILIAL, DUE TO LIGAND-DEFECTIVE APOLIPOPROTEIN B;;APOLIPOPROTEIN B-100, FAMILIAL DEFECTIVE |
| *APOB* | ENSG00000291544 | HYPOBETALIPOPROTEINEMIA, FAMILIAL, 1; FHBL1;;HYPOBETALIPOPROTEINEMIA, FAMILIAL; FHBL;;ACANTHOCYTOSIS WITH HYPOBETALIPOPROTEINEMIA;;HYPOBETALIPOPROTEINEMIA, NORMOTRIGLYCERIDEMIC LOW DENSITY LIPOPROTEIN CHOLESTEROL LEVEL QUANTITATIVE TRAIT LOCUS 4, IN /.../; LDLCQ4, INCLUDED |
| *APOB* | ENSG00000084674 | HYPERCHOLESTEROLEMIA, FAMILIAL, 2; FHCL2;;HYPERCHOLESTEROLEMIA, AUTOSOMAL DOMINANT, TYPE B;;APOLIPOPROTEIN B-100, FAMILIAL LIGAND-DEFECTIVE;;HYPERCHOLESTEROLEMIA, FAMILIAL, DUE TO LIGAND-DEFECTIVE APOLIPOPROTEIN B;;APOLIPOPROTEIN B-100, FAMILIAL DEFECTIVE |
| *APOB* | ENSG00000084674 | HYPOBETALIPOPROTEINEMIA, FAMILIAL, 1; FHBL1;;HYPOBETALIPOPROTEINEMIA, FAMILIAL; FHBL;;ACANTHOCYTOSIS WITH HYPOBETALIPOPROTEINEMIA;;HYPOBETALIPOPROTEINEMIA, NORMOTRIGLYCERIDEMIC LOW DENSITY LIPOPROTEIN CHOLESTEROL LEVEL QUANTITATIVE TRAIT LOCUS 4, IN /.../; LDLCQ4, INCLUDED |
| *ATP7B* | ENSG00000123191 | WILSON DISEASE; WND;;WD;;HEPATOLENTICULAR DEGENERATION |
| *BAG3* | ENSG00000151929 | MYOPATHY, MYOFIBRILLAR, 6; MFM6 |
| *BAG3* | ENSG00000151929 | CARDIOMYOPATHY, DILATED, 1HH; CMD1HH |
| *BMPR1A* | ENSG00000107779 | JUVENILE POLYPOSIS SYNDROME; JPS;;POLYPOSIS, JUVENILE INTESTINAL; PJI;;JUVENILE INTESTINAL POLYPOSIS; JIP;;POLYPOSIS, FAMILIAL, OF ENTIRE GASTROINTESTINAL TRACT JUVENILE POLYPOSIS OF STOMACH, INCLUDED;;JUVENILE POLYPOSIS COLI, INCLUDED |
| *BMPR1A* | ENSG00000107779 | POLYPOSIS SYNDROME, HEREDITARY MIXED, 2; HMPS2 |
| *BRCA1* | ENSG00000012048 | BREAST CANCER;;BREAST CANCER, FAMILIAL BREAST CANCER, FAMILIAL MALE, INCLUDED |
| *BRCA1* | ENSG00000012048 | BREAST-OVARIAN CANCER, FAMILIAL, SUSCEPTIBILITY TO, 1; BROVCA1;;HBOC1 BREAST CANCER, FAMILIAL, SUSCEPTIBILITY TO, 1, INCLUDED;;OVARIAN CANCER, FAMILIAL, SUSCEPTIBILITY TO, 1, INCLUDED |
| *BRCA1* | ENSG00000012048 | PANCREATIC CANCER, SUSCEPTIBILITY TO, 4; PNCA4 |
| *BRCA1* | ENSG00000012048 | FANCONI ANEMIA, COMPLEMENTATION GROUP S; FANCS |
| *BRCA2* | ENSG00000139618 | BREAST CANCER;;BREAST CANCER, FAMILIAL BREAST CANCER, FAMILIAL MALE, INCLUDED |
| *BRCA2* | ENSG00000139618 | MEDULLOBLASTOMA; MDB;;MEDULLOBLASTOMA PREDISPOSITION SYNDROME |
| *BRCA2* | ENSG00000139618 | PROSTATE CANCER |
| *BRCA2* | ENSG00000139618 | WILMS TUMOR 1; WT1;;NEPHROBLASTOMA |
| *BRCA2* | ENSG00000139618 | FANCONI ANEMIA, COMPLEMENTATION GROUP D1; FANCD1;;FAD1 |
| *BRCA2* | ENSG00000139618 | BREAST-OVARIAN CANCER, FAMILIAL, SUSCEPTIBILITY TO, 2; BROVCA2;;HBOC2 BREAST CANCER, FAMILIAL, SUSCEPTIBILITY TO, 2, INCLUDED;;OVARIAN CANCER, FAMILIAL, SUSCEPTIBILITY TO, 2, INCLUDED |
| *BRCA2* | ENSG00000139618 | GLIOMA SUSCEPTIBILITY 3; GLM3 |
| *BRCA2* | ENSG00000139618 | PANCREATIC CANCER, SUSCEPTIBILITY TO, 2;;PNCA2 |
| *BTD* | ENSG00000169814 | BIOTINIDASE DEFICIENCY;;BTD DEFICIENCY;;MULTIPLE CARBOXYLASE DEFICIENCY, LATE-ONSET;;MULTIPLE CARBOXYLASE DEFICIENCY, JUVENILE-ONSET |
| *CACNA1S* | ENSG00000081248 | HYPOKALEMIC PERIODIC PARALYSIS, TYPE 1; HOKPP1;;HYPOKALEMIC PERIODIC PARALYSIS; HOKPP |
| *CACNA1S* | ENSG00000081248 | THYROTOXIC PERIODIC PARALYSIS, SUSCEPTIBILITY TO, 1; TTPP1 |
| *CACNA1S* | ENSG00000081248 | MALIGNANT HYPERTHERMIA, SUSCEPTIBILITY TO, 5; MHS5 |
| *CACNA1S* | ENSG00000081248 | CONGENITAL MYOPATHY 18; CMYP18;;MYOPATHY, CONGENITAL, DUE TO DIHYDROPYRIDINE RECEPTOR DEFECT; DHPRM;;DIHYDROPYRIDINE RECEPTOR CONGENITAL MYOPATHY;;DHPR CONGENITAL MYOPATHY |
| *CALM1* | ENSG00000198668 | VENTRICULAR TACHYCARDIA, CATECHOLAMINERGIC POLYMORPHIC, 4; CPVT4 |
| *CALM1* | ENSG00000198668 | LONG QT SYNDROME 14; LQT14 |
| *CALM2* | ENSG00000143933 | LONG QT SYNDROME 15; LQT15 |
| *CALM3* | ENSG00000160014 | LONG QT SYNDROME 16; LQT16 VENTRICULAR TACHYCARDIA, CATECHOLAMINERGIC POLYMORPHIC 6, INCLUDED; CPVT6, INCLUDED |
| *CASQ2* | ENSG00000118729 | VENTRICULAR TACHYCARDIA, CATECHOLAMINERGIC POLYMORPHIC, 1, WITH OR WITHOUT ATRIAL DYSFUNCTION AND/OR DILATED CARDIOMYOPATHY; CPVT1;;VENTRICULAR TACHYCARDIA, STRESS-INDUCED POLYMORPHIC 1; VTSIP1;;VTSIP |
| *CASQ2* | ENSG00000118729 | VENTRICULAR TACHYCARDIA, CATECHOLAMINERGIC POLYMORPHIC, 2; CPVT2;;VENTRICULAR TACHYCARDIA, STRESS-INDUCED POLYMORPHIC 2; VTSIP2 |
| *COL3A1* | ENSG00000291748 | EHLERS-DANLOS SYNDROME, VASCULAR TYPE; EDSVASC;;EHLERS-DANLOS SYNDROME, TYPE IV, AUTOSOMAL DOMINANT; EDS4;;EDS IV;;EHLERS-DANLOS SYNDROME, ARTERIAL TYPE;;EHLERS-DANLOS SYNDROME, ECCHYMOTIC TYPE;;EHLERS-DANLOS SYNDROME, SACK-BARABAS TYPE |
| *COL3A1* | ENSG00000291748 | POLYMICROGYRIA WITH OR WITHOUT VASCULAR-TYPE EHLERS-DANLOS SYNDROME; PMGEDSV |
| *COL3A1* | ENSG00000168542 | EHLERS-DANLOS SYNDROME, VASCULAR TYPE; EDSVASC;;EHLERS-DANLOS SYNDROME, TYPE IV, AUTOSOMAL DOMINANT; EDS4;;EDS IV;;EHLERS-DANLOS SYNDROME, ARTERIAL TYPE;;EHLERS-DANLOS SYNDROME, ECCHYMOTIC TYPE;;EHLERS-DANLOS SYNDROME, SACK-BARABAS TYPE |
| *COL3A1* | ENSG00000168542 | POLYMICROGYRIA WITH OR WITHOUT VASCULAR-TYPE EHLERS-DANLOS SYNDROME; PMGEDSV |
| *DES* | ENSG00000175084 | SCAPULOPERONEAL SYNDROME, NEUROGENIC, KAESER TYPE; SCPNK;;KAESER SYNDROME;;STARK-KAESER SYNDROME;;SCAPULOPERONEAL SYNDROME, NEUROGENIC TYPE, OF KAESER |
| *DES* | ENSG00000175084 | MYOPATHY, MYOFIBRILLAR, 1; MFM1;;MYOPATHY, MYOFIBRILLAR, DESMIN-RELATED;;DESMINOPATHY, PRIMARY;;DESMIN-RELATED MYOPATHY; DRM;;MYOFIBRILLAR MYOPATHY WITH ARRHYTHMOGENIC RIGHT VENTRICULAR CARDIOMYOPATHY;;DESMIN-RELATED MYOPATHY WITH ARRHYTHMOGENIC RIG /.../TRICULAR CARDIOMYOPATHY;;ARRHYTHMOGENIC RIGHT VENTRICULAR DYSPLASIA, FAMILIAL, 7, FORMERLY; ARVD7, FORMERLY;;ARRHYTHMOGENIC RIGHT VENTRICULAR CARDIOMYOPATHY 7, FORMERLY; ARVC7, FORMERLY;;INCLUSION BODY MYOPATHY 1, AUTOSOMAL DOMINANT, FORMERLY; IBM1, FORMERLY;;CARDIOMYOPATHY, DILATED, 1F AND LIMB-GIRDLE MUSCULAR DYSTROPHY TYPE 1D, FORMERLY;;CMD1F AND LGMD1D, FORMERLY;;CARDIOMYOPATHY, DILATED, WITH CONDUCTION DEFECT AND MUSCULAR DYSTROPHY; CDCD3, FORMERLY;;MUSCULAR DYSTROPHY, LIMB-GIRDLE, TYPE 2R, FORMERLY; LGMD2R, FORMERLY |
| *DES* | ENSG00000175084 | CARDIOMYOPATHY, DILATED, 1I; CMD1I |
| *DSC2* | ENSG00000134755 | ARRHYTHMOGENIC RIGHT VENTRICULAR DYSPLASIA, FAMILIAL, 11; ARVD11;;ARRHYTHMOGENIC RIGHT VENTRICULAR CARDIOMYOPATHY 11; ARVC11 ARRHYTHMOGENIC RIGHT VENTRICULAR DYSPLASIA, FAMILIAL, 11, AND MILD PALMOPLANTAR KERATODERMA WITH OR WITHOUT WOOLLY HAIR, INCLUDED |
| *DSG2* | ENSG00000046604 | ARRHYTHMOGENIC RIGHT VENTRICULAR DYSPLASIA, FAMILIAL, 10; ARVD10;;ARRHYTHMOGENIC RIGHT VENTRICULAR CARDIOMYOPATHY 10; ARVC10 |
| *DSG2* | ENSG00000046604 | CARDIOMYOPATHY, DILATED, 1BB; CMD1BB |
| *DSP* | ENSG00000096696 | CARDIOMYOPATHY, DILATED, WITH WOOLLY HAIR AND KERATODERMA; DCWHK;;CARVAJAL SYNDROME;;PALMOPLANTAR KERATODERMA WITH LEFT VENTRICULAR CARDIOMYOPATHY AND WOOLLY HAIR |
| *DSP* | ENSG00000096696 | ARRHYTHMOGENIC RIGHT VENTRICULAR DYSPLASIA, FAMILIAL, 8; ARVD8;;ARRHYTHMOGENIC RIGHT VENTRICULAR CARDIOMYOPATHY 8; ARVC8 |
| *DSP* | ENSG00000096696 | EPIDERMOLYSIS BULLOSA, LETHAL ACANTHOLYTIC; EBLA;;LETHAL ACANTHOLYTIC EPIDERMOLYSIS BULLOSA; LAEB |
| *DSP* | ENSG00000096696 | KERATOSIS PALMOPLANTARIS STRIATA II; PPKS2;;KERATODERMA, PALMOPLANTAR, STRIATE FORM II; KPPS2;;STRIATE PALMOPLANTAR KERATODERMA II; SPPK2 |
| *DSP* | ENSG00000096696 | CARDIOMYOPATHY, DILATED, WITH WOOLLY HAIR, KERATODERMA, AND TOOTH AGENESIS; DCWHKTA |
| *ENG* | ENSG00000106991 | TELANGIECTASIA, HEREDITARY HEMORRHAGIC, TYPE 1; HHT1;;HHT;;TELANGIECTASIA, HEREDITARY HEMORRHAGIC, OF RENDU, OSLER, AND WEBER;;OSLER-RENDU-WEBER DISEASE;;ORW DISEASE |
| *FBN1* | ENSG00000166147 | ACROMICRIC DYSPLASIA; ACMICD |
| *FBN1* | ENSG00000166147 | ECTOPIA LENTIS 1, ISOLATED, AUTOSOMAL DOMINANT; ECTOL1 |
| *FBN1* | ENSG00000166147 | MARFAN SYNDROME; MFS;;MARFAN SYNDROME, TYPE I; MFS1 |
| *FBN1* | ENSG00000166147 | STIFF SKIN SYNDROME; SSKS |
| *FBN1* | ENSG00000166147 | MASS SYNDROME;;MASS PHENOTYPE;;OVERLAP CONNECTIVE TISSUE DISEASE; OCTD |
| *FBN1* | ENSG00000166147 | WEILL-MARCHESANI SYNDROME 2; WMS2;;WEILL-MARCHESANI SYNDROME, AUTOSOMAL DOMINANT;;SPHEROPHAKIA-BRACHYMORPHIA SYNDROME;;MESODERMAL DYSMORPHODYSTROPHY, CONGENITAL;;GLAUCOMA-LENS ECTOPIA-MICROSPHEROPHAKIA-STIFFNESS-SHORTNESS SYNDROME; GEMSS |
| *FBN1* | ENSG00000166147 | GELEOPHYSIC DYSPLASIA 2; GPHYSD2 |
| *FBN1* | ENSG00000166147 | MARFANOID-PROGEROID-LIPODYSTROPHY SYNDROME; MFLS;;MARFAN LIPODYSTROPHY SYNDROME;;MARFANOID-PROGEROID SYNDROME;;MARFAN-PROGEROID-LIPODYSTROPHY SYNDROME |
| *FLNC* | ENSG00000128591 | MYOPATHY, MYOFIBRILLAR, 5; MFM5;;MYOPATHY, MYOFIBRILLAR, FILAMIN C-RELATED;;FILAMINOPATHY, AUTOSOMAL DOMINANT |
| *FLNC* | ENSG00000128591 | MYOPATHY, DISTAL, 4; MPD4;;WILLIAMS DISTAL MYOPATHY |
| *FLNC* | ENSG00000128591 | CARDIOMYOPATHY, FAMILIAL HYPERTROPHIC, 26; CMH26 CARDIOMYOPATHY, FAMILIAL RESTRICTIVE, 5, INCLUDED; RCM5, INCLUDED;;CARDIOMYOPATHY, DILATED, 1PP, INCLUDED; CMD1PP, INCLUDED;;ARRHYTHMOGENIC RIGHT VENTRICULAR DYSPLASIA, FAMILIAL, 15, INCLUDED; ARVD15, /.../DED;;ARRHYTHMOGENIC RIGHT VENTRICULAR CARDIOMYOPATHY 15, INCLUDED; ARVC15, INCLUDED |
| *GAA* | ENSG00000291507 | GLYCOGEN STORAGE DISEASE II; GSD2;;GSD II;;ACID ALPHA-GLUCOSIDASE DEFICIENCY;;GAA DEFICIENCY;;POMPE DISEASE;;GLYCOGENOSIS, GENERALIZED, CARDIAC FORM;;CARDIOMEGALIA GLYCOGENICA DIFFUSA;;ACID MALTASE DEFICIENCY; AMD;;ALPHA-1,4-GLUCOSIDASE DEFICIENCY |
| *GAA* | ENSG00000171298 | GLYCOGEN STORAGE DISEASE II; GSD2;;GSD II;;ACID ALPHA-GLUCOSIDASE DEFICIENCY;;GAA DEFICIENCY;;POMPE DISEASE;;GLYCOGENOSIS, GENERALIZED, CARDIAC FORM;;CARDIOMEGALIA GLYCOGENICA DIFFUSA;;ACID MALTASE DEFICIENCY; AMD;;ALPHA-1,4-GLUCOSIDASE DEFICIENCY |
| *GLA* | ENSG00000102393 | FABRY DISEASE;;ANGIOKERATOMA CORPORIS DIFFUSUM;;ANDERSON-FABRY DISEASE;;HEREDITARY DYSTOPIC LIPIDOSIS;;ALPHA-GALACTOSIDASE A DEFICIENCY;;GLA DEFICIENCY;;CERAMIDE TRIHEXOSIDASE DEFICIENCY FABRY DISEASE, CARDIAC VARIANT, INCLUDED |
| *HFE* | ENSG00000010704 | HEMOCHROMATOSIS, TYPE 1; HFE1;;HEMOCHROMATOSIS; HFE;;HEMOCHROMATOSIS, HEREDITARY; HH |
| *HNF1A* | ENSG00000135100 | TYPE 2 DIABETES MELLITUS; T2D;;DIABETES MELLITUS, NONINSULIN-DEPENDENT; NIDDM;;NONINSULIN-DEPENDENT DIABETES MELLITUS;;DIABETES MELLITUS, TYPE II;;MATURITY-ONSET DIABETES INSULIN RESISTANCE, SUSCEPTIBILITY TO, INCLUDED;;DIABETES MELLITUS, TYPE 2, PR /.../ON AGAINST, INCLUDED |
| *HNF1A* | ENSG00000135100 | HEPATIC ADENOMAS, FAMILIAL;;LIVER CELL ADENOMAS, FAMILIAL |
| *HNF1A* | ENSG00000135100 | RENAL CELL CARCINOMA, NONPAPILLARY; RCC;;HYPERNEPHROMA;;ADENOCARCINOMA OF KIDNEY NONPAPILLARY RENAL CARCINOMA 1 LOCUS, INCLUDED; NRC1, INCLUDED |
| *HNF1A* | ENSG00000135100 | TYPE 1 DIABETES MELLITUS; T1D;;DIABETES MELLITUS, INSULIN-DEPENDENT; IDDM;;JUVENILE-ONSET DIABETES; JOD TYPE 1 DIABETES MELLITUS 1, INCLUDED; T1D1, INCLUDED;;DIABETES MELLITUS, INSULIN-DEPENDENT, 1, INCLUDED; IDDM1, INCLUDED;;INSULIN-DEPENDENT DIABE /.../LLITUS 1, INCLUDED |
| *HNF1A* | ENSG00000135100 | MATURITY-ONSET DIABETES OF THE YOUNG, TYPE 3; MODY3;;MODY, TYPE 3 |
| *HNF1A* | ENSG00000135100 | TYPE 1 DIABETES MELLITUS 20; T1D20;;DIABETES MELLITUS, INSULIN-DEPENDENT, 20; IDDM20 |
| *KCNH2* | ENSG00000055118 | SHORT QT SYNDROME 1; SQT1 |
| *KCNH2* | ENSG00000055118 | LONG QT SYNDROME 2; LQT2 LONG QT SYNDROME 1/2, DIGENIC, INCLUDED; LQT1/2, DIGENIC, INCLUDED;;LONG QT SYNDROME 2/3, DIGENIC, INCLUDED; LQT2/3, DIGENIC, INCLUDED;;LONG QT SYNDROME 2/5, DIGENIC, INCLUDED; LQT2/5, DIGENIC, INCLUDED;;LONG QT SYNDROME 2/9 /.../NIC, INCLUDED; LQT2/9, DIGENIC, INCLUDED |
| *KCNQ1* | ENSG00000282076 | BECKWITH-WIEDEMANN SYNDROME; BWS;;EXOMPHALOS-MACROGLOSSIA-GIGANTISM SYNDROME;;EMG SYNDROME;;WIEDEMANN-BECKWITH SYNDROME; WBS BECKWITH-WIEDEMANN SYNDROME CHROMOSOME REGION, INCLUDED; BWCR, INCLUDED |
| *KCNQ1* | ENSG00000282076 | LONG QT SYNDROME 1; LQT1;;WARD-ROMANO SYNDROME; WRS;;ROMANO-WARD SYNDROME; RWS;;VENTRICULAR FIBRILLATION WITH PROLONGED QT INTERVAL LONG QT SYNDROME 1/2, DIGENIC, INCLUDED; LQT1/2, DIGENIC, INCLUDED;;LONG QT SYNDROME 1, ACQUIRED, SUSCEPTIBILITY TO, /.../ED |
| *KCNQ1* | ENSG00000282076 | JERVELL AND LANGE-NIELSEN SYNDROME 1; JLNS1;;DEAFNESS, CONGENITAL, AND FUNCTIONAL HEART DISEASE;;PROLONGED QT INTERVAL IN EKG AND SUDDEN DEATH;;CARDIOAUDITORY SYNDROME OF JERVELL AND LANGE-NIELSEN;;SURDO-CARDIAC SYNDROME |
| *KCNQ1* | ENSG00000282076 | ATRIAL FIBRILLATION, FAMILIAL, 3; ATFB3 |
| *KCNQ1* | ENSG00000282076 | SHORT QT SYNDROME 2; SQT2 |
| *KCNQ1* | ENSG00000053918 | BECKWITH-WIEDEMANN SYNDROME; BWS;;EXOMPHALOS-MACROGLOSSIA-GIGANTISM SYNDROME;;EMG SYNDROME;;WIEDEMANN-BECKWITH SYNDROME; WBS BECKWITH-WIEDEMANN SYNDROME CHROMOSOME REGION, INCLUDED; BWCR, INCLUDED |
| *KCNQ1* | ENSG00000053918 | LONG QT SYNDROME 1; LQT1;;WARD-ROMANO SYNDROME; WRS;;ROMANO-WARD SYNDROME; RWS;;VENTRICULAR FIBRILLATION WITH PROLONGED QT INTERVAL LONG QT SYNDROME 1/2, DIGENIC, INCLUDED; LQT1/2, DIGENIC, INCLUDED;;LONG QT SYNDROME 1, ACQUIRED, SUSCEPTIBILITY TO, /.../ED |
| *KCNQ1* | ENSG00000053918 | JERVELL AND LANGE-NIELSEN SYNDROME 1; JLNS1;;DEAFNESS, CONGENITAL, AND FUNCTIONAL HEART DISEASE;;PROLONGED QT INTERVAL IN EKG AND SUDDEN DEATH;;CARDIOAUDITORY SYNDROME OF JERVELL AND LANGE-NIELSEN;;SURDO-CARDIAC SYNDROME |
| *KCNQ1* | ENSG00000053918 | ATRIAL FIBRILLATION, FAMILIAL, 3; ATFB3 |
| *KCNQ1* | ENSG00000053918 | SHORT QT SYNDROME 2; SQT2 |
| *LDLR* | ENSG00000130164 | HYPERCHOLESTEROLEMIA, FAMILIAL, 1; FHCL1;;FHC; FH;;HYPERLIPOPROTEINEMIA, TYPE II;;HYPERLIPOPROTEINEMIA, TYPE IIA;;HYPER-LOW-DENSITY-LIPOPROTEINEMIA;;HYPERCHOLESTEROLEMIC XANTHOMATOSIS, FAMILIAL;;LDL RECEPTOR DISORDER LOW DENSITY LIPOPROTEIN CHOLESTE /.../VEL QUANTITATIVE TRAIT LOCUS 2, INCLUDED; LDLCQ2, INCLUDED |
| *LMNA* | ENSG00000160789 | CARDIOMYOPATHY, DILATED, 1A; CMD1A;;CARDIOMYOPATHY, DILATED, WITH CONDUCTION DEFECT 1; CDCD1;;CARDIOMYOPATHY, IDIOPATHIC DILATED;;CARDIOMYOPATHY, FAMILIAL IDIOPATHIC;;CARDIOMYOPATHY, CONGESTIVE |
| *LMNA* | ENSG00000160789 | LIPODYSTROPHY, FAMILIAL PARTIAL, TYPE 2; FPLD2;;FPL2;;LIPODYSTROPHY, FAMILIAL PARTIAL, DUNNIGAN TYPE;;LIPODYSTROPHY, FAMILIAL, OF LIMBS AND LOWER TRUNK;;LIPODYSTROPHY, REVERSE PARTIAL;;LIPOATROPHIC DIABETES |
| *LMNA* | ENSG00000160789 | HUTCHINSON-GILFORD PROGERIA SYNDROME; HGPS;;PROGERIA PROGERIA SYNDROME, CHILDHOOD-ONSET, INCLUDED |
| *LMNA* | ENSG00000160789 | EMERY-DREIFUSS MUSCULAR DYSTROPHY 2, AUTOSOMAL DOMINANT; EDMD2;;EMD2;;EMERY-DREIFUSS MUSCULAR DYSTROPHY, AUTOSOMAL DOMINANT;;SCAPULOILIOPERONEAL ATROPHY WITH CARDIOPATHY;;MUSCULAR DYSTROPHY WITH EARLY CONTRACTURES AND CARDIOMYOPATHY, AUTOSOMAL DOMIN /.../AUPTMANN-THANNHAUSER MUSCULAR DYSTROPHY;;CARDIOMYOPATHY, DILATED, WITH QUADRICEPS MYOPATHY;;MUSCULAR DYSTROPHY, LIMB-GIRDLE, TYPE 1B, FORMERLY; LGMD1B, FORMERLY;;MUSCULAR DYSTROPHY, PROXIMAL, TYPE 1B, FORMERLY |
| *LMNA* | ENSG00000160789 | CARDIOMYOPATHY, DILATED, WITH HYPERGONADOTROPIC HYPOGONADISM;;MALOUF SYNDROME;;CARDIOMYOPATHY, CONGESTIVE, WITH HYPERGONADOTROPIC HYPOGONADISM;;CARDIOMYOPATHY, DILATED, WITH PREMATURE OVARIAN FAILURE;;CARDIOMYOPATHY WITH PRIMARY TESTICULAR FAILURE;; /.../ SYNDROME;;GENITAL ANOMALY WITH CARDIOMYOPATHY;;CARDIOGENITAL SYNDROME |
| *LMNA* | ENSG00000160789 | MANDIBULOACRAL DYSPLASIA WITH TYPE A LIPODYSTROPHY; MADA;;LIPODYSTROPHY, TYPE A, ASSOCIATED WITH MANDIBULOACRAL DYSPLASIA;;CRANIOMANDIBULAR DERMATODYSOSTOSIS MANDIBULOACRAL DYSPLASIA WITH TYPE A LIPODYSTROPHY, ATYPICAL, INCLUDED |
| *LMNA* | ENSG00000160789 | CHARCOT-MARIE-TOOTH DISEASE, AXONAL, TYPE 2B1; CMT2B1;;CHARCOT-MARIE-TOOTH DISEASE, NEURONAL, TYPE 2B1;;CHARCOT-MARIE-TOOTH DISEASE, AXONAL, AUTOSOMAL RECESSIVE, TYPE 2B1;;CHARCOT-MARIE-TOOTH NEUROPATHY, TYPE 2B1 |
| *LMNA* | ENSG00000160789 | HEART-HAND SYNDROME, SLOVENIAN TYPE |
| *LMNA* | ENSG00000160789 | MUSCULAR DYSTROPHY, CONGENITAL, LMNA-RELATED;;MDCL |
| *LMNA* | ENSG00000160789 | EMERY-DREIFUSS MUSCULAR DYSTROPHY 3, AUTOSOMAL RECESSIVE; EDMD3 |
| *LMNA* | ENSG00000160789 | RESTRICTIVE DERMOPATHY 2; RSDM2;;RESTRICTIVE DERMOPATHY 2, LETHAL |
| *MAX* | ENSG00000125952 | PHEOCHROMOCYTOMA;;PHEOCHROMOCYTOMA, SUSCEPTIBILITY TO |
| *MAX* | ENSG00000125952 | POLYDACTYLY-MACROCEPHALY SYNDROME; PDMCS |
| *MEN1* | ENSG00000133895 | MULTIPLE ENDOCRINE NEOPLASIA, TYPE I; MEN1;;MEN I;;ENDOCRINE ADENOMATOSIS, MULTIPLE;;MEA I;;WERMER SYNDROME MEN1 SOMATIC MUTATIONS, INCLUDED |
| *MLH1* | ENSG00000076242 | MUIR-TORRE SYNDROME; MRTES;;CUTANEOUS SEBACEOUS NEOPLASMS AND KERATOACANTHOMAS, MULTIPLE, WITH GASTROINTESTINAL AND OTHER CARCINOMAS |
| *MLH1* | ENSG00000076242 | MISMATCH REPAIR CANCER SYNDROME 1; MMRCS1;;CONSTITUTIONAL MISMATCH REPAIR DEFICIENCY SYNDROME; CMMRDS;;MISMATCH REPAIR DEFICIENCY;;MMR DEFICIENCY;;CHILDHOOD CANCER SYNDROME;;BRAIN TUMOR-POLYPOSIS SYNDROME 1; BTPS1;;BTP1 SYNDROME;;TURCOT SYNDROME |
| *MLH1* | ENSG00000076242 | LYNCH SYNDROME 2; LYNCH2;;COLORECTAL CANCER, HEREDITARY NONPOLYPOSIS, TYPE 2; HNPCC2;;COLON CANCER, FAMILIAL NONPOLYPOSIS, TYPE 2; FCC2;;COCA2 |
| *MSH2* | ENSG00000095002 | LYNCH SYNDROME 1; LYNCH1;;COLORECTAL CANCER, HEREDITARY NONPOLYPOSIS, TYPE 1; HNPCC1;;COLON CANCER, FAMILIAL NONPOLYPOSIS, TYPE 1; FCC1;;COCA1;;LYNCH SYNDROME I, FORMERLY;;LYNCH SYNDROME II, FORMERLY |
| *MSH2* | ENSG00000095002 | MUIR-TORRE SYNDROME; MRTES;;CUTANEOUS SEBACEOUS NEOPLASMS AND KERATOACANTHOMAS, MULTIPLE, WITH GASTROINTESTINAL AND OTHER CARCINOMAS |
| *MSH2* | ENSG00000095002 | MISMATCH REPAIR CANCER SYNDROME 2; MMRCS2 |
| *MSH6* | ENSG00000116062 | ENDOMETRIAL CANCER |
| *MSH6* | ENSG00000116062 | LYNCH SYNDROME 5; LYNCH5;;COLORECTAL CANCER, HEREDITARY NONPOLYPOSIS, TYPE 5; HNPCC5 |
| *MSH6* | ENSG00000116062 | MISMATCH REPAIR CANCER SYNDROME 3; MMRCS3 |
| *MUTYH* | ENSG00000132781 | FAMILIAL ADENOMATOUS POLYPOSIS 2; FAP2;;COLORECTAL ADENOMATOUS POLYPOSIS, AUTOSOMAL RECESSIVE;;ADENOMAS, MULTIPLE COLORECTAL, AUTOSOMAL RECESSIVE |
| *MUTYH* | ENSG00000132781 | GASTRIC CANCER GASTRIC CANCER, INTESTINAL, INCLUDED |
| *MYBPC3* | ENSG00000134571 | CARDIOMYOPATHY, FAMILIAL HYPERTROPHIC, 4; CMH4 CARDIOMYOPATHY, FAMILIAL HYPERTROPHIC, 4, SUSCEPTIBILITY TO, INCLUDED |
| *MYBPC3* | ENSG00000134571 | LEFT VENTRICULAR NONCOMPACTION 10; LVNC10 CARDIOMYOPATHY, DILATED, 1MM, INCLUDED; CMD1MM, INCLUDED |
| *MYH11* | ENSG00000276480 | AORTIC ANEURYSM, FAMILIAL THORACIC 4; AAT4;;FAA4;;AORTIC ANEURYSM/AORTIC DISSECTION AND PATENT DUCTUS ARTERIOSUS |
| *MYH11* | ENSG00000276480 | VISCERAL MYOPATHY 2; VSCM2 |
| *MYH11* | ENSG00000276480 | MEGACYSTIS-MICROCOLON-INTESTINAL HYPOPERISTALSIS SYNDROME 2; MMIHS2 |
| *MYH11* | ENSG00000133392 | AORTIC ANEURYSM, FAMILIAL THORACIC 4; AAT4;;FAA4;;AORTIC ANEURYSM/AORTIC DISSECTION AND PATENT DUCTUS ARTERIOSUS |
| *MYH11* | ENSG00000133392 | VISCERAL MYOPATHY 2; VSCM2 |
| *MYH11* | ENSG00000133392 | MEGACYSTIS-MICROCOLON-INTESTINAL HYPOPERISTALSIS SYNDROME 2; MMIHS2 |
| *MYH7* | ENSG00000092054 | MYOPATHY, DISTAL, 1; MPD1;;MYOPATHY, LATE DISTAL HEREDITARY;;LAING DISTAL MYOPATHY;;MYOPATHY, DISTAL, EARLY-ONSET, AUTOSOMAL DOMINANT |
| *MYH7* | ENSG00000092054 | CARDIOMYOPATHY, FAMILIAL HYPERTROPHIC, 1; CMH1;;CMH;;VENTRICULAR HYPERTROPHY, HEREDITARY;;ASYMMETRIC SEPTAL HYPERTROPHY; ASH;;HYPERTROPHIC SUBAORTIC STENOSIS, IDIOPATHIC |
| *MYH7* | ENSG00000092054 | CONGENITAL MYOPATHY 7B, MYOSIN STORAGE, AUTOSOMAL RECESSIVE; CMYP7B;;MYOPATHY, MYOSIN STORAGE, AUTOSOMAL RECESSIVE; MSMB;;MYOPATHY, HYALINE BODY, AUTOSOMAL RECESSIVE |
| *MYH7* | ENSG00000092054 | CONGENITAL MYOPATHY 7A, MYOSIN STORAGE, AUTOSOMAL DOMINANT; CMYP7A;;MYOPATHY, MYOSIN STORAGE, AUTOSOMAL DOMINANT; MSMA;;MYOPATHY, HYALINE BODY, AUTOSOMAL DOMINANT;;MYOPATHY WITH LYSIS OF TYPE I MYOFIBRILS;;SCAPULOPERONEAL MYOPATHY, MYH7-RELATED; SPM /.../PULOPERONEAL MUSCULAR DYSTROPHY; SPMD;;SCAPULOPERONEAL SYNDROME, MYOPATHIC TYPE |
| *MYH7* | ENSG00000092054 | CARDIOMYOPATHY, DILATED, 1S; CMD1S LEFT VENTRICULAR NONCOMPACTION 5, INCLUDED; LVNC5, INCLUDED |
| *MYL2* | ENSG00000111245 | CARDIOMYOPATHY, FAMILIAL HYPERTROPHIC, 10; CMH10;;CARDIOMYOPATHY, HYPERTROPHIC, MID-LEFT VENTRICULAR CHAMBER TYPE, 2 |
| *MYL2* | ENSG00000111245 | MYOPATHY, MYOFIBRILLAR, 12, INFANTILE-ONSET, WITH CARDIOMYOPATHY; MFM12 |
| *MYL3* | ENSG00000160808 | CARDIOMYOPATHY, FAMILIAL HYPERTROPHIC, 8; CMH8;;CARDIOMYOPATHY, HYPERTROPHIC, MID-LEFT VENTRICULAR CHAMBER TYPE, 1 |
| *NF2* | ENSG00000186575 | SCHWANNOMATOSIS, VESTIBULAR; SWNV;;SCHWANNOMATOSIS 3; SWN3;;NEUROFIBROMATOSIS, TYPE II; NF2;;NEUROFIBROMATOSIS, CENTRAL TYPE;;ACOUSTIC SCHWANNOMAS, BILATERAL, FORMERLY;;BILATERAL ACOUSTIC NEUROFIBROMATOSIS, FORMERLY; BANF, FORMERLY;;ACOUSTIC NEURINO /.../LATERAL, FORMERLY; ACN, FORMERLY |
| *NF2* | ENSG00000186575 | MENINGIOMA, FAMILIAL, SUSCEPTIBILITY TO |
| *OTC* | ENSG00000036473 | ORNITHINE TRANSCARBAMYLASE DEFICIENCY, HYPERAMMONEMIA DUE TO;;ORNITHINE CARBAMOYLTRANSFERASE DEFICIENCY;;OTC DEFICIENCY |
| *PALB2* | ENSG00000083093 | FANCONI ANEMIA, COMPLEMENTATION GROUP N; FANCN |
| *PALB2* | ENSG00000083093 | PANCREATIC CANCER, SUSCEPTIBILITY TO, 3;;PNCA3 |
| *PALB2* | ENSG00000083093 | BREAST-OVARIAN CANCER, FAMILIAL, SUSCEPTIBILITY TO, 5; BROVCA5 |
| *PCSK9* | ENSG00000169174 | HYPERCHOLESTEROLEMIA, FAMILIAL, 3; FHCL3;;HYPERCHOLESTEROLEMIA, AUTOSOMAL DOMINANT, 3; HCHOLA3;;FH3 LOW DENSITY LIPOPROTEIN CHOLESTEROL LEVEL QUANTITATIVE TRAIT LOCUS 1, INCLUDED; LDLCQ1, INCLUDED |
| *PKP2* | ENSG00000057294 | ARRHYTHMOGENIC RIGHT VENTRICULAR DYSPLASIA, FAMILIAL, 9; ARVD9;;ARRHYTHMOGENIC RIGHT VENTRICULAR CARDIOMYOPATHY 9; ARVC9 |
| *PMS2* | ENSG00000122512 | LYNCH SYNDROME 4; LYNCH4;;COLORECTAL CANCER, HEREDITARY NONPOLYPOSIS, TYPE 4; HNPCC4 |
| *PMS2* | ENSG00000122512 | MISMATCH REPAIR CANCER SYNDROME 4; MMRCS4 |
| *PRKAG2* | ENSG00000106617 | WOLFF-PARKINSON-WHITE SYNDROME; WPW;;WPW SYNDROME PREEXCITATION SYNDROME, INCLUDED;;ACCESSORY ATRIOVENTRICULAR PATHWAYS, INCLUDED |
| *PRKAG2* | ENSG00000106617 | GLYCOGEN STORAGE DISEASE OF HEART, LETHAL CONGENITAL;;PHOSPHORYLASE KINASE DEFICIENCY OF HEART;;GLYCOGEN STORAGE DISEASE OF HEART |
| *PRKAG2* | ENSG00000106617 | CARDIOMYOPATHY, FAMILIAL HYPERTROPHIC, 6; CMH6 |
| *PTEN* | ENSG00000284792 | COWDEN SYNDROME 1; CWS1;;CS; CD;;MULTIPLE HAMARTOMA SYNDROME; MHAM;;PTEN HAMARTOMA TUMOR SYNDROME; PHTS;;PTEN HAMARTOMA TUMOR SYNDROME WITH GRANULAR CELL TUMOR;;BANNAYAN-RILEY-RUVALCABA SYNDROME; BBRS;;BANNAYAN-ZONANA SYNDROME; BZS;;RILEY-SMITH SYND /.../RUVALCABA-MYHRE-SMITH SYNDROME; RMSS;;MACROCEPHALY, PSEUDOPAPILLEDEMA, AND MULTIPLE HEMANGIOMATA;;MACROCEPHALY, MULTIPLE LIPOMAS, AND HEMANGIOMATA LHERMITTE-DUCLOS DISEASE, INCLUDED; LDD, INCLUDED;;DYSPLASTIC GANGLIOCYTOMA OF THE CEREBELLUM, INCLUDED;;CEREBELLOPARENCHYMAL DISORDER VI, INCLUDED; CPD6, INCLUDED;;CEREBELLAR GRANULE CELL HYPERTROPHY AND MEGALENCEPHALY, INCLUDED;;PROTEUS-LIKE SYNDROME, INCLUDED |
| *PTEN* | ENSG00000284792 | PROSTATE CANCER |
| *PTEN* | ENSG00000284792 | MACROCEPHALY/AUTISM SYNDROME |
| *PTEN* | ENSG00000284792 | MENINGIOMA, FAMILIAL, SUSCEPTIBILITY TO |
| *PTEN* | ENSG00000284792 | GLIOMA SUSCEPTIBILITY 2; GLM2 |
| *PTEN* | ENSG00000171862 | COWDEN SYNDROME 1; CWS1;;CS; CD;;MULTIPLE HAMARTOMA SYNDROME; MHAM;;PTEN HAMARTOMA TUMOR SYNDROME; PHTS;;PTEN HAMARTOMA TUMOR SYNDROME WITH GRANULAR CELL TUMOR;;BANNAYAN-RILEY-RUVALCABA SYNDROME; BBRS;;BANNAYAN-ZONANA SYNDROME; BZS;;RILEY-SMITH SYND /.../RUVALCABA-MYHRE-SMITH SYNDROME; RMSS;;MACROCEPHALY, PSEUDOPAPILLEDEMA, AND MULTIPLE HEMANGIOMATA;;MACROCEPHALY, MULTIPLE LIPOMAS, AND HEMANGIOMATA LHERMITTE-DUCLOS DISEASE, INCLUDED; LDD, INCLUDED;;DYSPLASTIC GANGLIOCYTOMA OF THE CEREBELLUM, INCLUDED;;CEREBELLOPARENCHYMAL DISORDER VI, INCLUDED; CPD6, INCLUDED;;CEREBELLAR GRANULE CELL HYPERTROPHY AND MEGALENCEPHALY, INCLUDED;;PROTEUS-LIKE SYNDROME, INCLUDED |
| *PTEN* | ENSG00000171862 | PROSTATE CANCER |
| *PTEN* | ENSG00000171862 | MACROCEPHALY/AUTISM SYNDROME |
| *PTEN* | ENSG00000171862 | MENINGIOMA, FAMILIAL, SUSCEPTIBILITY TO |
| *PTEN* | ENSG00000171862 | GLIOMA SUSCEPTIBILITY 2; GLM2 |
| *RB1* | ENSG00000139687 | BLADDER CANCER |
| *RB1* | ENSG00000139687 | RETINOBLASTOMA; RB1;;RB |
| *RB1* | ENSG00000139687 | SMALL CELL CANCER OF THE LUNG;;SCLC1;;SCLC; SCCL |
| *RB1* | ENSG00000139687 | OSTEOGENIC SARCOMA;;OSTEOSARCOMA; OSRC |
| *RBM20* | ENSG00000203867 | CARDIOMYOPATHY, DILATED, 1DD; CMD1DD |
| *RET* | ENSG00000165731 | HIRSCHSPRUNG DISEASE, SUSCEPTIBILITY TO, 1; HSCR1;;HIRSCHSPRUNG DISEASE; HSCR;;AGANGLIONIC MEGACOLON;;MEGACOLON, AGANGLIONIC; MGC HIRSCHSPRUNG DISEASE, PROTECTION AGAINST, INCLUDED |
| *RET* | ENSG00000165731 | THYROID CARCINOMA, FAMILIAL MEDULLARY; MTC;;FMTC;;MTC1 |
| *RET* | ENSG00000165731 | MULTIPLE ENDOCRINE NEOPLASIA, TYPE IIB; MEN2B;;MEN IIB;;NEUROMATA, MUCOSAL, WITH ENDOCRINE TUMORS;;WAGENMANN-FROBOESE SYNDROME;;MULTIPLE ENDOCRINE NEOPLASIA, TYPE III, FORMERLY; MEN3, FORMERLY MUCOSAL NEUROMA SYNDROME, INCLUDED |
| *RET* | ENSG00000165731 | PHEOCHROMOCYTOMA;;PHEOCHROMOCYTOMA, SUSCEPTIBILITY TO |
| *RET* | ENSG00000165731 | MULTIPLE ENDOCRINE NEOPLASIA, TYPE IIA; MEN2A;;PHEOCHROMOCYTOMA AND AMYLOID-PRODUCING MEDULLARY THYROID CARCINOMA;;PTC SYNDROME;;SIPPLE SYNDROME THYROID CARCINOMA, FAMILIAL MEDULLARY, INCLUDED |
| *RPE65* | ENSG00000116745 | LEBER CONGENITAL AMAUROSIS 2; LCA2;;AMAUROSIS CONGENITA OF LEBER II |
| *RPE65* | ENSG00000116745 | RETINITIS PIGMENTOSA 20; RP20 |
| *RPE65* | ENSG00000116745 | RETINITIS PIGMENTOSA 87 WITH CHOROIDAL INVOLVEMENT; RP87 |
| *RYR1* | ENSG00000196218 | CONGENITAL MYOPATHY 1A, AUTOSOMAL DOMINANT, WITH SUSCEPTIBILITY TO MALIGNANT HYPERTHERMIA; CMYP1A;;CENTRAL CORE DISEASE OF MUSCLE; CCD;;CCO |
| *RYR1* | ENSG00000196218 | MALIGNANT HYPERTHERMIA, SUSCEPTIBILITY TO, 1; MHS1;;MHS;;HYPERTHERMIA OF ANESTHESIA;;HYPERPYREXIA, MALIGNANT; MH |
| *RYR1* | ENSG00000196218 | CONGENITAL MYOPATHY 1B, AUTOSOMAL RECESSIVE; CMYP1B;;MINICORE MYOPATHY WITH EXTERNAL OPHTHALMOPLEGIA;;MINICORE MYOPATHY;;MULTICORE MYOPATHY;;MULTIMINICORE MYOPATHY;;MULTICORE MYOPATHY WITH EXTERNAL OPHTHALMOPLEGIA;;MULTIMINICORE DISEASE WITH EXTERNA /.../HALMOPLEGIA |
| *RYR1* | ENSG00000196218 | KING-DENBOROUGH SYNDROME; KDS;;KING SYNDROME |
| *RYR2* | ENSG00000198626 | VENTRICULAR ARRHYTHMIAS DUE TO CARDIAC RYANODINE RECEPTOR CALCIUM RELEASE DEFICIENCY SYNDROME; VACRDS;;RYR2 CALCIUM RELEASE DEFICIENCY SYNDROME |
| *RYR2* | ENSG00000198626 | VENTRICULAR TACHYCARDIA, CATECHOLAMINERGIC POLYMORPHIC, 1, WITH OR WITHOUT ATRIAL DYSFUNCTION AND/OR DILATED CARDIOMYOPATHY; CPVT1;;VENTRICULAR TACHYCARDIA, STRESS-INDUCED POLYMORPHIC 1; VTSIP1;;VTSIP |
| *SCN5A* | ENSG00000183873 | PROGRESSIVE FAMILIAL HEART BLOCK, TYPE IA; PFHB1A;;PFHBIA;;HEART BLOCK, PROGRESSIVE FAMILIAL, TYPE I; PFHBI;;LENEGRE-LEV DISEASE;;CARDIAC CONDUCTION DEFECT, PROGRESSIVE; PCCD;;BUNDLE BRANCH BLOCK;;HEREDITARY BUNDLE BRANCH SYSTEM DEFECT; HBBD HEART B /.../NONPROGRESSIVE, INCLUDED;;CARDIAC CONDUCTION DEFECT, NONPROGRESSIVE, INCLUDED |
| *SCN5A* | ENSG00000183873 | SUDDEN INFANT DEATH SYNDROME;;SIDS |
| *SCN5A* | ENSG00000183873 | BRUGADA SYNDROME 1; BRGDA1;;RIGHT BUNDLE BRANCH BLOCK, ST SEGMENT ELEVATION, AND SUDDEN DEATH SYNDROME;;SUDDEN UNEXPLAINED NOCTURNAL DEATH SYNDROME; SUNDS CARDIAC CONDUCTION DEFECT, NONSPECIFIC, INCLUDED |
| *SCN5A* | ENSG00000183873 | CARDIOMYOPATHY, DILATED, 1E; CMD1E;;CARDIOMYOPATHY, DILATED, WITH CONDUCTION DISORDER AND ARRHYTHMIA;;CARDIOMYOPATHY, DILATED, WITH CONDUCTION DEFECT 2; CDCD2 |
| *SCN5A* | ENSG00000183873 | VENTRICULAR FIBRILLATION, PAROXYSMAL FAMILIAL, 1; VF1;;VF;;IVF VENTRICULAR FIBRILLATION DURING MYOCARDIAL INFARCTION, SUSCEPTIBILITY TO, INCLUDED |
| *SCN5A* | ENSG00000183873 | LONG QT SYNDROME 3; LQT3 LONG QT SYNDROME 3, ACQUIRED, SUSCEPTIBILITY TO, INCLUDED;;LONG QT SYNDROME 2/3, DIGENIC, INCLUDED; LQT2/3, DIGENIC, INCLUDED;;LONG QT SYNDROME 3/6, DIGENIC, INCLUDED; LQT3/6, DIGENIC, INCLUDED |
| *SCN5A* | ENSG00000183873 | SICK SINUS SYNDROME 1; SSS1;;SINUS RHYTHM, CONGENITAL ABSENCE OF;;SINUS NODE DISEASE, FAMILIAL, AUTOSOMAL RECESSIVE;;SICK SINUS SYNDROME, CONGENITAL;;SINUS BRADYCARDIA SYNDROME, FAMILIAL |
| *SCN5A* | ENSG00000183873 | ATRIAL FIBRILLATION, FAMILIAL, 10; ATFB10 |
| *SDHAF2* | ENSG00000167985 | PHEOCHROMOCYTOMA/PARAGANGLIOMA SYNDROME 2; PPGL2;;PARAGANGLIOMAS 2; PGL2;;GLOMUS TUMORS, FAMILIAL, 2 |
| *SDHB* | ENSG00000117118 | PHEOCHROMOCYTOMA/PARAGANGLIOMA SYNDROME 4; PPGL4;;PARAGANGLIOMAS 4; PGL4;;CAROTID BODY TUMORS AND MULTIPLE EXTRAADRENAL PHEOCHROMOCYTOMAS;;PHEOCHROMOCYTOMA, EXTRAADRENAL, AND CERVICAL PARAGANGLIOMA;;PARAGANGLIOMAS, HEREDITARY EXTRAADRENAL;;PHEOCHROM /.../A, FAMILIAL EXTRAADRENAL;;PARAGANGLIOMA, FAMILIAL MALIGNANT |
| *SDHB* | ENSG00000117118 | GASTROINTESTINAL STROMAL TUMOR; GIST |
| *SDHB* | ENSG00000117118 | PARAGANGLIOMA AND GASTRIC STROMAL SARCOMA;;CARNEY-STRATAKIS SYNDROME;;PARAGANGLIOMA AND GASTROINTESTINAL STROMAL TUMOR |
| *SDHB* | ENSG00000117118 | MITOCHONDRIAL COMPLEX II DEFICIENCY, NUCLEAR TYPE 4; MC2DN4 |
| *SDHC* | ENSG00000143252 | PHEOCHROMOCYTOMA/PARAGANGLIOMA SYNDROME 3; PPGL3;;PARAGANGLIOMAS 3; PGL3;;GLOMUS TUMORS, FAMILIAL, 3 |
| *SDHC* | ENSG00000143252 | GASTROINTESTINAL STROMAL TUMOR; GIST |
| *SDHC* | ENSG00000143252 | PARAGANGLIOMA AND GASTRIC STROMAL SARCOMA;;CARNEY-STRATAKIS SYNDROME;;PARAGANGLIOMA AND GASTROINTESTINAL STROMAL TUMOR |
| *SDHD* | ENSG00000204370 | PHEOCHROMOCYTOMA/PARAGANGLIOMA SYNDROME 1; PPGL1;;PARAGANGLIOMAS 1; PGL1;;PARAGANGLIOMAS, FAMILIAL, 1;;PARAGANGLIOMATA; PGL;;GLOMUS TUMORS, FAMILIAL, 1;;CHEMODECTOMAS;;CAROTID BODY TUMORS; CBT1;;GLOMUS JUGULARE TUMORS;;PARAGANGLIOMA, CAROTID BODY;;P /.../GLIOMAS, FAMILIAL NONCHROMAFFIN, 1 |
| *SDHD* | ENSG00000204370 | PARAGANGLIOMA AND GASTRIC STROMAL SARCOMA;;CARNEY-STRATAKIS SYNDROME;;PARAGANGLIOMA AND GASTROINTESTINAL STROMAL TUMOR |
| *SDHD* | ENSG00000204370 | MITOCHONDRIAL COMPLEX II DEFICIENCY, NUCLEAR TYPE 3; MC2DN3 |
| *SMAD3* | ENSG00000166949 | LOEYS-DIETZ SYNDROME 3; LDS3;;ANEURYSMS-OSTEOARTHRITIS SYNDROME;;LOEYS-DIETZ SYNDROME WITH OSTEOARTHRITIS;;LOEYS-DIETZ SYNDROME, TYPE 1C, FORMERLY; LDS1C, FORMERLY |
| *SMAD4* | ENSG00000141646 | MYHRE SYNDROME; MYHRS;;LARYNGOTRACHEAL STENOSIS, ARTHROPATHY, PROGNATHISM, AND SHORT STATURE;;LAPS SYNDROME;;GROWTH-MENTAL DEFICIENCY SYNDROME OF MYHRE |
| *SMAD4* | ENSG00000141646 | JUVENILE POLYPOSIS SYNDROME; JPS;;POLYPOSIS, JUVENILE INTESTINAL; PJI;;JUVENILE INTESTINAL POLYPOSIS; JIP;;POLYPOSIS, FAMILIAL, OF ENTIRE GASTROINTESTINAL TRACT JUVENILE POLYPOSIS OF STOMACH, INCLUDED;;JUVENILE POLYPOSIS COLI, INCLUDED |
| *SMAD4* | ENSG00000141646 | JUVENILE POLYPOSIS/HEREDITARY HEMORRHAGIC TELANGIECTASIA SYNDROME; JPHT;;JP/HHT SYNDROME;;JUVENILE POLYPOSIS WITH HEREDITARY HEMORRHAGIC TELANGIECTASIA;;JPS/HHT;;TELANGIECTASIA, HEREDITARY HEMORRHAGIC, WITH JUVENILE POLYPOSIS COLI;;POLYPOSIS, GENERA /.../JUVENILE, WITH PULMONARY ARTERIOVENOUS MALFORMATION |
| *SMAD4* | ENSG00000141646 | PANCREATIC CANCER;;PANCREATIC CARCINOMA;;PANCREATIC ACINAR CARCINOMA |
| *STK11* | ENSG00000118046 | MELANOMA, CUTANEOUS MALIGNANT, SUSCEPTIBILITY TO, 1; CMM1;;MELANOMA, CUTANEOUS MALIGNANT; CMM;;MELANOMA, MALIGNANT;;FAMILIAL ATYPICAL MOLE-MALIGNANT MELANOMA SYNDROME; FAMMM;;MELANOMA, FAMILIAL; MLM;;DYSPLASTIC NEVUS SYNDROME, HEREDITARY; DNS;;B-K M /.../NDROME |
| *STK11* | ENSG00000118046 | PEUTZ-JEGHERS SYNDROME; PJS;;POLYPOSIS, HAMARTOMATOUS INTESTINAL;;POLYPS-AND-SPOTS SYNDROME |
| *STK11* | ENSG00000118046 | PANCREATIC CANCER;;PANCREATIC CARCINOMA;;PANCREATIC ACINAR CARCINOMA |
| *STK11* | ENSG00000118046 | TESTICULAR GERM CELL TUMOR; TGCT;;MALE GERM CELL TUMOR; MGCT SEMINOMA, INCLUDED;;NONSEMINOMATOUS GERM CELL TUMORS, INCLUDED;;TERATOMA, TESTICULAR, INCLUDED;;EMBRYONAL CELL CARCINOMA, INCLUDED;;ENDODERMAL SINUS TUMOR, INCLUDED;;SPERMATOCYTIC SEMINOMA /.../UDED |
| *TGFBR1* | ENSG00000106799 | MULTIPLE SELF-HEALING SQUAMOUS EPITHELIOMA, SUSCEPTIBILITY TO; MSSE;;FERGUSON-SMITH-TYPE EPITHELIOMA;;ESS1, FORMERLY |
| *TGFBR1* | ENSG00000106799 | LOEYS-DIETZ SYNDROME 1; LDS1;;FURLONG SYNDROME;;LOEYS-DIETZ AORTIC ANEURYSM SYNDROME;;AORTIC ANEURYSM, FAMILIAL THORACIC 5; AAT5 |
| *TGFBR2* | ENSG00000163513 | ESOPHAGEAL CANCER ESOPHAGEAL SQUAMOUS CELL CARCINOMA, SUSCEPTIBILITY TO, INCLUDED;;ESCC, SUSCEPTIBILITY TO, INCLUDED;;AERODIGESTIVE TRACT CANCER, SUSCEPTIBILITY TO, INCLUDED;;GASTRIC CARDIA ADENOCARCINOMA, SUSCEPTIBILITY TO, INCLUDED |
| *TGFBR2* | ENSG00000163513 | LOEYS-DIETZ SYNDROME 2; LDS2;;AORTIC ANEURYSM, FAMILIAL THORACIC 3; AAT3;;MARFAN SYNDROME, TYPE II, FORMERLY |
| *TGFBR2* | ENSG00000163513 | COLORECTAL CANCER, HEREDITARY NONPOLYPOSIS, TYPE 6; HNPCC6;;COLON CANCER, HEREDITARY NONPOLYPOSIS, TYPE 6 |
| *TMEM127* | ENSG00000135956 | PHEOCHROMOCYTOMA;;PHEOCHROMOCYTOMA, SUSCEPTIBILITY TO |
| *TMEM43* | ENSG00000170876 | ARRHYTHMOGENIC RIGHT VENTRICULAR DYSPLASIA, FAMILIAL, 5; ARVD5;;ARRHYTHMOGENIC RIGHT VENTRICULAR CARDIOMYOPATHY 5; ARVC5 |
| *TMEM43* | ENSG00000170876 | EMERY-DREIFUSS MUSCULAR DYSTROPHY 7, AUTOSOMAL DOMINANT; EDMD7 |
| *TMEM43* | ENSG00000170876 | AUDITORY NEUROPATHY, AUTOSOMAL DOMINANT 3; AUNA3 |
| *TNNC1* | ENSG00000114854 | CARDIOMYOPATHY, DILATED, 1Z; CMD1Z |
| *TNNC1* | ENSG00000114854 | CARDIOMYOPATHY, FAMILIAL HYPERTROPHIC, 13; CMH13 |
| *TNNI3* | ENSG00000129991 | CARDIOMYOPATHY, FAMILIAL RESTRICTIVE, 1; RCM1;;RCM |
| *TNNI3* | ENSG00000129991 | CARDIOMYOPATHY, DILATED, 2A; CMD2A;;CARDIOMYOPATHY, DILATED, AUTOSOMAL RECESSIVE;;CARDIOMYOPATHY, CONGESTIVE, AUTOSOMAL RECESSIVE |
| *TNNI3* | ENSG00000129991 | CARDIOMYOPATHY, DILATED, 1FF; CMD1FF |
| *TNNI3* | ENSG00000129991 | CARDIOMYOPATHY, FAMILIAL HYPERTROPHIC, 7; CMH7 |
| *TNNT2* | ENSG00000118194 | CARDIOMYOPATHY, FAMILIAL HYPERTROPHIC, 2; CMH2 |
| *TNNT2* | ENSG00000118194 | CARDIOMYOPATHY, DILATED, 1D; CMD1D;;LEFT VENTRICULAR NONCOMPACTION 6, INCLUDED; LVNC6, INCLUDED |
| *TNNT2* | ENSG00000118194 | CARDIOMYOPATHY, FAMILIAL RESTRICTIVE, 3; RCM3 |
| *TP53* | ENSG00000141510 | BREAST CANCER;;BREAST CANCER, FAMILIAL BREAST CANCER, FAMILIAL MALE, INCLUDED |
| *TP53* | ENSG00000141510 | COLORECTAL CANCER; CRC;;COLON CANCER |
| *TP53* | ENSG00000141510 | HEPATOCELLULAR CARCINOMA;;HCC;;CANCER, HEPATOCELLULAR;;LIVER CANCER;;LIVER CELL CARCINOMA; LCC;;HEPATOMA HEPATOBLASTOMA, INCLUDED;;HEPATOBLASTOMA CAUSED BY SOMATIC MUTATION, INCLUDED |
| *TP53* | ENSG00000141510 | GLIOMA SUSCEPTIBILITY 1; GLM1 GLIOMA OF BRAIN, FAMILIAL, INCLUDED; GLM, INCLUDED;;GLIOBLASTOMA MULTIFORME, INCLUDED; GBM, INCLUDED;;ASTROCYTOMA, INCLUDED;;OLIGODENDROGLIOMA, INCLUDED;;EPENDYMOMA, INCLUDED;;SUBEPENDYMOMA, INCLUDED |
| *TP53* | ENSG00000141510 | LI-FRAUMENI SYNDROME; LFS;;SARCOMA FAMILY SYNDROME OF LI AND FRAUMENI;;SBLA SYNDROME LI-FRAUMENI-LIKE SYNDROME, INCLUDED; LFL, INCLUDED |
| *TP53* | ENSG00000141510 | ADRENOCORTICAL CARCINOMA, HEREDITARY; ADCC ADRENOCORTICAL CARCINOMA, PEDIATRIC, INCLUDED |
| *TP53* | ENSG00000141510 | OSTEOGENIC SARCOMA;;OSTEOSARCOMA; OSRC |
| *TP53* | ENSG00000141510 | PANCREATIC CANCER;;PANCREATIC CARCINOMA;;PANCREATIC ACINAR CARCINOMA |
| *TP53* | ENSG00000141510 | PAPILLOMA OF CHOROID PLEXUS; CPP;;CHOROID PLEXUS PAPILLOMA CHOROID PLEXUS CARCINOMA, INCLUDED; CPC, INCLUDED |
| *TP53* | ENSG00000141510 | NASOPHARYNGEAL CARCINOMA;;NPCA;;NPC;;NASOPHARYNGEAL CANCER NASOPHARYNGEAL CARCINOMA, SUSCEPTIBILITY TO, 1, INCLUDED; NPCA1, INCLUDED |
| *TP53* | ENSG00000141510 | BASAL CELL CARCINOMA, SUSCEPTIBILITY TO, 7; BCC7 |
| *TP53* | ENSG00000141510 | BONE MARROW FAILURE SYNDROME 5; BMFS5 |
| *TPM1* | ENSG00000140416 | CARDIOMYOPATHY, FAMILIAL HYPERTROPHIC, 3; CMH3 |
| *TPM1* | ENSG00000140416 | CARDIOMYOPATHY, DILATED, 1Y; CMD1Y LEFT VENTRICULAR NONCOMPACTION 9, INCLUDED; LVNC9, INCLUDED |
| *TRDN* | ENSG00000186439 | VENTRICULAR TACHYCARDIA, CATECHOLAMINERGIC POLYMORPHIC, 1, WITH OR WITHOUT ATRIAL DYSFUNCTION AND/OR DILATED CARDIOMYOPATHY; CPVT1;;VENTRICULAR TACHYCARDIA, STRESS-INDUCED POLYMORPHIC 1; VTSIP1;;VTSIP |
| *TRDN* | ENSG00000186439 | CARDIAC ARRHYTHMIA SYNDROME, WITH OR WITHOUT SKELETAL MUSCLE WEAKNESS; CARDAR;;TRIADEN KNOCKOUT SYNDROME;;VENTRICULAR TACHYCARDIA, CATECHOLAMINERGIC POLYMORPHIC, 5, WITH OR WITHOUT MUSCLE WEAKNESS; CPVT5 |
| *TSC1* | ENSG00000165699 | TUBEROUS SCLEROSIS 1; TSC1;;TUBEROUS SCLEROSIS COMPLEX; TSC;;TUBEROSE SCLEROSIS; TS |
| *TSC1* | ENSG00000165699 | LYMPHANGIOLEIOMYOMATOSIS; LAM;;LYMPHANGIOMYOMATOSIS |
| *TSC1* | ENSG00000165699 | FOCAL CORTICAL DYSPLASIA, TYPE II; FCORD2;;CORTICAL DYSPLASIA OF TAYLOR; CDT;;FOCAL CORTICAL DYSPLASIA OF TAYLOR; FCDT;;FCD2 FOCAL CORTICAL DYSPLASIA, TYPE IIA, INCLUDED; FCORD2A, INCLUDED;;FCD IIA, INCLUDED;;CORTICAL DYSPLASIA OF TAYLOR WITHOUT BAL /.../ELLS, INCLUDED;;CORTICAL DYSPLASIA OF TAYLOR, DYSPLASIA ONLY, INCLUDED; CDTD, INCLUDED;;FOCAL CORTICAL DYSPLASIA, TYPE IIB, INCLUDED; FCORD2B, INCLUDED;;FCD IIB, INCLUDED;;CORTICAL DYSPLASIA OF TAYLOR WITH BALLOON CELLS, INCLUDED; CDTBC, INCLUDED |
| *TSC2* | ENSG00000103197 | LYMPHANGIOLEIOMYOMATOSIS; LAM;;LYMPHANGIOMYOMATOSIS |
| *TSC2* | ENSG00000103197 | FOCAL CORTICAL DYSPLASIA, TYPE II; FCORD2;;CORTICAL DYSPLASIA OF TAYLOR; CDT;;FOCAL CORTICAL DYSPLASIA OF TAYLOR; FCDT;;FCD2 FOCAL CORTICAL DYSPLASIA, TYPE IIA, INCLUDED; FCORD2A, INCLUDED;;FCD IIA, INCLUDED;;CORTICAL DYSPLASIA OF TAYLOR WITHOUT BAL /.../ELLS, INCLUDED;;CORTICAL DYSPLASIA OF TAYLOR, DYSPLASIA ONLY, INCLUDED; CDTD, INCLUDED;;FOCAL CORTICAL DYSPLASIA, TYPE IIB, INCLUDED; FCORD2B, INCLUDED;;FCD IIB, INCLUDED;;CORTICAL DYSPLASIA OF TAYLOR WITH BALLOON CELLS, INCLUDED; CDTBC, INCLUDED |
| *TSC2* | ENSG00000103197 | TUBEROUS SCLEROSIS 2; TSC2 TSC2 ANGIOMYOLIPOMAS, RENAL, MODIFIER OF, INCLUDED |
| *TTN* | ENSG00000155657 | TIBIAL MUSCULAR DYSTROPHY, TARDIVE; TMD;;TARDIVE TIBIAL MUSCULAR DYSTROPHY;;UDD MYOPATHY |
| *TTN* | ENSG00000155657 | MYOPATHY, MYOFIBRILLAR, 9, WITH EARLY RESPIRATORY FAILURE; MFM9;;HEREDITARY MYOPATHY WITH EARLY RESPIRATORY FAILURE; HMERF;;MYOPATHY, PROXIMAL, WITH EARLY RESPIRATORY MUSCLE INVOLVEMENT; MPRM;;EDSTROM MYOPATHY;;MYOPATHY, DISTAL, WITH EARLY RESPIRATO /.../LURE, AUTOSOMAL DOMINANT |
| *TTN* | ENSG00000155657 | CARDIOMYOPATHY, DILATED, 1G; CMD1G |
| *TTN* | ENSG00000155657 | MUSCULAR DYSTROPHY, LIMB-GIRDLE, AUTOSOMAL RECESSIVE 10; LGMDR10;;MUSCULAR DYSTROPHY, LIMB-GIRDLE, TYPE 2J; LGMD2J |
| *TTN* | ENSG00000155657 | CONGENITAL MYOPATHY 5 WITH CARDIOMYOPATHY; CMYP5;;SALIH MYOPATHY; SALMY;;MYOPATHY, EARLY-ONSET, WITH FATAL CARDIOMYOPATHY; EOMFC |
| *TTN* | ENSG00000155657 | CARDIOMYOPATHY, FAMILIAL HYPERTROPHIC, 9; CMH9 |
| *TTR* | ENSG00000118271 | AMYLOIDOSIS, HEREDITARY, TRANSTHYRETIN-RELATED;;HEREDITARY AMYLOIDOSIS, TRANSTHYRETIN-RELATED;;TRANSTHYRETIN AMYLOIDOSIS;;AMYLOID POLYNEUROPATHY, FAMILIAL; FAP AMYLOIDOSIS, LEPTOMENINGEAL, TRANSTHYRETIN-RELATED, INCLUDED;;AMYLOID CARDIOMYOPATHY, TRA /.../ETIN-RELATED, INCLUDED |
| *TTR* | ENSG00000118271 | CARPAL TUNNEL SYNDROME 1; CTS1 CARPAL TUNNEL SYNDROME, INCLUDED; CTS, INCLUDED;;AMYOTROPHY, THENAR, OF CARPAL ORIGIN, INCLUDED |
| *TTR* | ENSG00000118271 | HYPERTHYROXINEMIA, DYSTRANSTHYRETINEMIC; DTTRH;;HYPERTHYROXINEMIA, DYSPREALBUMINEMIC;;DYSTRANSTHYRETINEMIC EUTHYROIDAL HYPERTHYROXINEMIA;;EUTHRYROIDAL HYPERTHYROXINEMIA 2 |
| *VHL* | ENSG00000134086 | RENAL CELL CARCINOMA, NONPAPILLARY; RCC;;HYPERNEPHROMA;;ADENOCARCINOMA OF KIDNEY NONPAPILLARY RENAL CARCINOMA 1 LOCUS, INCLUDED; NRC1, INCLUDED |
| *VHL* | ENSG00000134086 | PHEOCHROMOCYTOMA;;PHEOCHROMOCYTOMA, SUSCEPTIBILITY TO |
| *VHL* | ENSG00000134086 | VON HIPPEL-LINDAU SYNDROME; VHLS;;VHL VON HIPPEL-LINDAU SYNDROME, MODIFIERS OF, INCLUDED |
| *VHL* | ENSG00000134086 | ERYTHROCYTOSIS, FAMILIAL, 2; ECYT2;;POLYCYTHEMIA, VHL-DEPENDENT POLYCYTHEMIA, CHUVASH TYPE, INCLUDED |
| *WT1* | ENSG00000184937 | ANIRIDIA 1; AN1;;AN;;ANIRIDIA II, FORMERLY; AN2, FORMERLY CATARACT, CONGENITAL, WITH LATE-ONSET CORNEAL DYSTROPHY, INCLUDED |
| *WT1* | ENSG00000184937 | FRASIER SYNDROME |
| *WT1* | ENSG00000184937 | MESOTHELIOMA, MALIGNANT; MESOM |
| *WT1* | ENSG00000184937 | WILMS TUMOR 1; WT1;;NEPHROBLASTOMA |
| *WT1* | ENSG00000184937 | WILMS TUMOR, ANIRIDIA, GENITOURINARY ANOMALIES, AND IMPAIRED INTELLECTUAL DEVELOPMENT SYNDROME; WAGR;;WILMS TUMOR, ANIRIDIA, GENITOURINARY ANOMALIES, AND MENTAL RETARDATION SYNDROME;;WAGR SYNDROME;;CHROMOSOME 11p13 DELETION SYNDROME |
| *WT1* | ENSG00000184937 | DENYS-DRASH SYNDROME; DDS;;DRASH SYNDROME;;WILMS TUMOR AND PSEUDO- OR TRUE HERMAPHRODITISM;;NEPHROPATHY, WILMS TUMOR, AND GENITAL ANOMALIES |
| *WT1* | ENSG00000184937 | NEPHROTIC SYNDROME, TYPE 4; NPHS4 |
| *WT1* | ENSG00000184937 | MEACHAM SYNDROME |

**Supplementary table 2: The in-silico predicted impact of coding substitutions on protein stability and assessment of evolutionary conservation**

| **Gene** | **AA Change** | **Conservation** | **Stability change ΔΔG (kcal/mol)** | **CADD** | **AlphaMissense** | **EVE** | **ESM-1b** |
| --- | --- | --- | --- | --- | --- | --- | --- |
| *KCNQ1* | p.Arg397Trp | 0.45 moderate | 0.22 unlikely to be destabilising | 24.2 | 0.27 benign | 0.35 benign | -13.4 pathogenic |
| *DES* | p.Arg350Trp | 0.97 very high | 0.57 unlikely to be destabilising | 28.0 | 0.44 ambiguous | 0.84 pathogenic | -11.8 pathogenic |
| *RYR1* | p.Arg530His | 1.00 very high | 0.86 unlikely to be destabilising | 32.0 | 0.49 ambiguous |  | -11.8 pathogenic |
| *MYBPC3* | p.Gly531Arg | 0.99 | 3.38 likely to be destabilising | 23.5 | 0.24 benign | 0.80 pathogenic | -11.3 pathogenic |
| *MSH2* | p.Leu341Val | 0.90 | 1.63 unlikely to be destabilising | 23.3 | 0.20 benign | 0.72 pathogenic | -11.2 pathogenic |
| *FBN1* | p.Arg165Gln | 0.78 | 0.98 unlikely to be destabilising | 29.7 | 0.35 ambiguous |  | -8.1 uncertain |
| *RYR1* | p.Asp3501Tyr | 0.98 |  | 29.5 | 0.83 pathogenic | 0.53 uncertain | -16.4 pathogenic |
